# The Pros and Cons of Estrogens in Prostate Cancer: An Update with a Focus on Phytoestrogens

**DOI:** 10.3390/biomedicines12081636

**Published:** 2024-07-23

**Authors:** Marília I. Figueira, Tiago M. A. Carvalho, Joana Macário-Monteiro, Henrique J. Cardoso, Sara Correia, Cátia V. Vaz, Ana P. Duarte, Sílvia Socorro

**Affiliations:** CICS-UBI, Centro de Investigação em Ciências da Saúde, Universidade da Beira Interior, 6200-506 Covilhã, Portugal; minf91@gmail.com (M.I.F.);

**Keywords:** prostate cancer, estrogens, phytoestrogens, estrogen receptors, GPER, daidzein, genistein, quercetin, coumestrol, resveratrol

## Abstract

The role of estrogens in prostate cancer (PCa) is shrouded in mystery, with its actions going from angelic to devilish. The findings by Huggins and Hodges establishing PCa as a hormone-sensitive cancer have provided the basis for using estrogens in therapy. However, despite the clinical efficacy in suppressing tumor growth and the panoply of experimental evidence describing its anticarcinogenic effects, estrogens were abolished from PCa treatment because of the adverse secondary effects. Notwithstanding, research work over the years has continued investigating the effects of estrogens, reporting their pros and cons in prostate carcinogenesis. In contrast with the beneficial therapeutic effects, many reports have implicated estrogens in the disruption of prostate cell fate and tissue homeostasis. On the other hand, epidemiological data demonstrating the lower incidence of PCa in Eastern countries associated with a higher consumption of phytoestrogens support the beneficial role of estrogens in counteracting cancer development. Many studies have investigated the effects of phytoestrogens and the underlying mechanisms of action, which may contribute to developing safe estrogen-based anti-PCa therapies. This review compiles the existing data on the anti- and protumorigenic actions of estrogens and summarizes the anticancer effects of several phytoestrogens, highlighting their promising features in PCa treatment.

## 1. Introduction

Estrogens are steroid hormones mainly associated with female reproductive physiology. Nevertheless, this class of sex hormones is involved in a panoply of biological processes in both men and women [1,2,3]. Regardless of sex, estrone (E_1_), 17b-estradiol (E_2_), and estriol (E_3_) are the primary naturally occurring estrogens, with E_2_ being the most potent and prevalent [4,5]. The pioneering work of Huggins and Hodges [6] in the middle of the 20th century has provided the basis for the use of hormones in prostate cancer (PCa) therapy by establishing PCa as a hormone-sensitive cancer, stimulated by androgenic activity and inhibited by the suppression of androgen levels or estrogen administration. In fact, estrogens were used in PCa treatment between the 1940s and 1970s, with the synthetic estrogen diethylstilbestrol (DES) being an effective therapy for delaying the progression of metastatic PCa [7,8,9,10,11,12]. However, after decades of practice, estrogen therapy was discontinued due to the associated adverse effects, namely at the cardiovascular level [8,9,10].

The withdrawal of estrogens from clinical practice did not eliminate the interest in these hormones, and research efforts continued deepening the understanding of estrogenic effects in the prostate. The pros and cons of the actions of estrogens in the regulation of prostate cell fate and targeting the different cancer hallmarks have been reported over the years. In parallel to the beneficial effects in suppressing tumor growth, accumulating evidence has pointed to estrogens as causative agents of PCa, promoting cell survival and malignant transformation, leading to tumor growth and metastization [13,14,15].

An extensive amount of research has been carried out to understand the duality of estrogen’s actions in PCa. Although the detailed mechanisms are far from being completely clarified, the double-edged sword of estrogen effects has been highly linked to the separate and distinct activity of estrogen receptors (ERs), which has been elegantly reviewed in the past years [16,17,18]. At a glance, estrogen actions are mediated by the classical nuclear ERs, namely ERα and ERβ, and the membrane-bound G protein-coupled estrogen receptor (GPER) [19,20,21]. ERα and ERβ act as ligand-activated transcription factors exerting direct effects on the regulation of gene expression by binding to specific DNA sequences in the target genes (genomic signaling) [22,23], or alternatively, by interacting with other transcription factors or activating intracellular signaling cascades, indirectly affecting gene expression [24,25]. GPER is involved in rapid nongenomic estrogenic responses through the activation of signal-transduction mechanisms with the subsequent production of intracellular second messengers, cAMP regulation, and protein-kinase activation in signaling cascades, which can ultimately also lead to indirect gene expression regulation [19,20,21,26].

It has been widely accepted that ERα and ERβ drive differential responses in PCa [27,28], with ERα associated with protumorigenic effects, whereas ERβ has been implicated in the antitumorigenic actions of estrogens [29,30,31]. The complexity of estrogen actions in PCa increases considering GPER, as this membrane receptor has been shown to trigger both tumor growth and tumor regression effects [18]. Overall, despite disclosing the mechanism of action and the panoply of anticancer actions, research efforts have not found safe approaches for re-introducing estrogens in PCa therapy [32].

In line with the beneficial effects of estrogens in PCa treatment are the epidemiological studies describing the lower incidence of PCa in Eastern countries associated with a plant-based diet and a higher consumption of phytoestrogens [33,34,35], which have raised curiosity about the therapeutic potential of these compounds. At present, important lines of research have explored the use of phytoestrogens and bioactive compounds with estrogen-like activity in PCa treatment [36]. Several compounds, alone or in combined approaches, have shown promising results in controlling PCa development and progression without adverse effects [37,38,39,40,41,42,43], and have been tested in preclinical studies and clinical trials as possible estrogen therapies [44,45,46,47,48,49]. The present review revisits the effects of natural and synthetic estrogens as anticancer molecules and, opposingly, their actions in fueling PCa hallmarks such as cell survival, proliferative activity, resistance to apoptotic cell death, and invasiveness. Additionally, the actions of diverse natural compounds with estrogenic activity and promising anticancer effects are summarized. Our main goal was to compile the current information, fulfilling the scientific gaps existing in reviewing the subject. The reviews published in the last three years have only focused on one type of ER, a specific class of compounds, or stage of PCa, or more generally, have covered several types of cancer simultaneously, without exhaustively detailing the effects of estrogens in PCa. Herein, an integrated and translational perspective regarding estrogen actions in PCa is provided, and the possible contribution of phytoestrogens to therapy is discussed.

## 2. Estrogens in the Male

### 2.1. Circulating Levels

The measurement methodology is the first issue to consider in any analysis of the systematized steroid circulating levels. Among the different methods that can be used to quantify steroid levels in human blood samples, estrogens have essentially been measured by radioimmunoassay (RIA), chemiluminescence immunoassays, and liquid chromatography tandem mass spectrometry (LC-MS/MS), which have the advantage of detecting small amounts of steroids, thus reaching lower limits of quantification. Therefore, several authors have indicated LC-MS/MS as the preferred methodology for quantifying estrogen serum levels [50,51], though RIA remains the most commonly used method (Table 1).

Another important aspect in comparing studies is the units of measurement of the serum steroid concentrations, which also depends on the methodology used. The reported E_2_ measurements were converted to molar concentrations (nM) to allow for immediate comparisons (Table 1).

Data collected from the literature and summarized in Table 1 show that a broad range of serum E_2_ concentrations can be found in healthy men, from 0.028 to 0.235 nM. Overall, very low levels were obtained from healthy individuals between 30 and 90 years old. This broad age range encompasses the critical age for the development of PCa, which is 50 years old and above [52]. Moreover, the widespread values reported for E_2_ serum concentrations could be linked to the age group and the ethnicity or physiological conditions. Indeed, several endocrine and metabolic events, namely the increase in adiposity and body mass index, with the augmented extragonadal aromatization of testosterone, could influence the E_2_ levels [53,54,55]. Furthermore, circulating estrogens appeared to be inversely associated with intraprostatic inflammation [56]. However, these factors were not always considered in the available studies, mainly when comparing healthy men and PCa patients.

The relationship between PCa and E_2_ serum levels has been largely controversial, and the majority of studies have not found statistically significant associations between hormone concentrations and the risk of PCa [57,58,59]. Thee E_2_ serum levels found in PCa patients were also highly variable, ranging from 0.050 to 0.300 nM (Table 1). Although not consensual, some studies have reported a slightly significant increase in E_2_ levels in PCa cases (Table 1) [55,60,61,62].

**Table 1:** E_2_ serum levels in healthy men and PCa patients.

E_2_ Concentration Range (nM)		Methodology of Measurement	Ref.
Healthy Men	PCa Patients		
0.156	0.200 *	RIA	[61]
0.110–0.160	0.120–0.160	RIA	[63]
0.114–0.125	0.110–0.128 *	Dextran-coated charcoal method	[55]
0.110–0.160	0.120–0.160	RIA	[63]
0.200	-	RIA	[64]
0.093	-	Chemiluminescence immunoassay	[65]
0.200	-	RIA	[66]
0.066–0.221	0.066–0.233	-	[67]
0.125	0.121	RIA	[68]
0.070	-	Chemiluminescence immunoassay	[69]
0.100–0.150	0.050–0.300	RIA	[70]
0.235	0.247	-	[71]
0.089–0.120	0.086–0.106	Gas chromatography-mass spectrometry	[54]
0.062	0.066	RIA	[72]
0.028–0.167	-	Chemiluminescence immunoassay	[73]
0.106	0.106	RIA	[74]
0.122	0.122	Heterogeneous competitive magnetic separation assay	[75]
0.028–0.156	0.063–0.068	Chemiluminescence immunoassay	[76]
0.107	0.101–0.105	Chemiluminescence immunoassay	[77]
0.082–0.234	0.200	-	[50]
0.108	0.159 *	Enzyme linked immunosorbent assay (ELISA)	[62]
0.132	-	RIA	[78]
0.103	-	RIA	[79]

* statistically significant difference.

### 2.2. Intraprostatic Production

**Figure 1:**
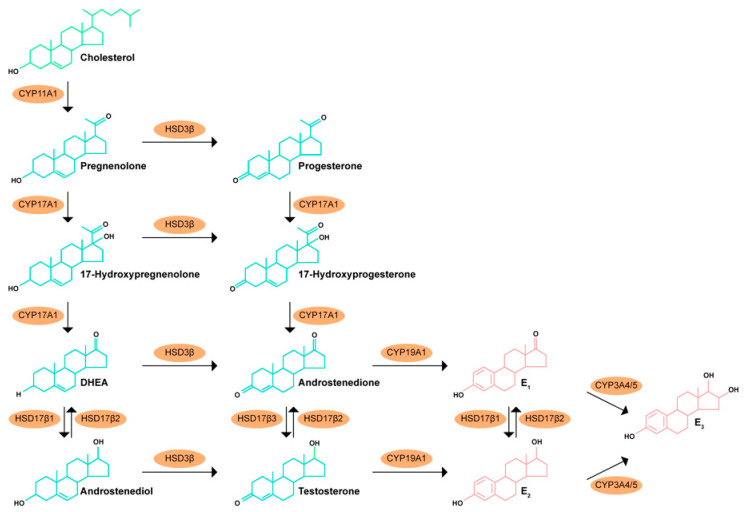
Estrogen biosynthesis pathway. Cholesterol is the precursor of steroid hormones. It originates pregnenolone through the cholesterol side-chain cleavage enzyme (CYP11A1), which is converted to progesterone by 3β-hydroxysteroid dehydrogenase (HSD3β). This enzyme is also responsible for the conversion of 17-hydroxypregnenolone to 17-hydroxyprogesterone, dehydroepiandrosterone (DHEA) to androstenedione, and androstenediol to testosterone. Estrogens are produced through the activity of aromatase (CYP19A1), which converts androstenedione and testosterone to estrone (E1) and 17β-estradiol (E2), respectively. E_1_ is reversibly converted to E_2_ by the enzyme 17β-hydroxysteroid dehydrogenase (HSD17β1/2), and both can originate E_3_ through the cytochrome P450 3A4/5 (CYP3A4/5).

Estrogen biosynthesis is the last reaction on the steroidogenic pathway, and it occurs due to the activity of the heme protein aromatase/P450aro/CYP19A1 enzyme [80,81]. Aromatase in the endoplasmic reticulum [82] or mitochondria [83] catalyzes the irreversible aromatization of the A ring of the androgen precursors, generating estrogens. The naturally occurring estrogens E_1_, E_2_, and E_3_ (Figure 1) are produced via the aromatization of androstenedione and testosterone [5]. E_1_ can also be reversibly converted to E_2_ by the enzyme 17β-hydroxysteroid dehydrogenase, and both E_1_ and E_2_ are precursors of E_3_ through the involvement of CYP3A4/5 [84].

The aromatization of androgens in the male occurs mainly in the somatic cells of the testis and other peripheral organs including the prostate [85,86,87]. Prostate cells have been shown to express a functional aromatase that uses external androgen precursors such as dehydroepiandrosterone (DHEA), dehydroepiandrosterone sulfate (DHEA-S), and circulating testosterone to produce estrogenic compounds (Figure 1) [80,88].

Estrogen synthesis in the human prostate was first pointed out by Matzin and Soloway [86] based on the immunohistochemical localization of aromatase in prostatic tissues. This was followed by the characterization of aromatase expression in both the epithelial and stromal cells of the human prostate [87], and later on by the demonstration that prostatic stromal cells can synthesize E_2_ from testosterone [89]. Over the years, the local production of estrogens in the prostate and its functional relevance have been studied. Manipulating the aromatase expression levels or activity has been shown to directly affect intraprostatic E_2_ levels. Moreover, the localization of aromatase in the prostate cells is “conveniently” adjacent to ERs, the mediators of the actions of estrogens in regulating prostate physiology [90,91]. Upregulation of aromatase activity increased the intracellular levels of estrogens in prostate cells concomitantly with ERα and GPER activation [85,92]. In contrast, upon aromatase inhibition, intraprostatic E_2_ levels were significantly reduced [93].

Altered intraprostatic estrogen biosynthesis has been reported in the neoplastic prostate. Results from the STERKPROSER trial suggest that human cancerous prostates have a higher activity of aromatase, producing more E_2_ compared with the noncancerous prostate tissues [60]. Moreover, an approximately 30-fold increase in aromatase expression was found in PCa metastatic tissues compared to primary tumors [94].

## 3. Estrogens in Prostate Cancer Therapy

### 3.1. Old-Times and Withdrawal

The use of estrogens in PCa therapy goes back to the 1940s. The pioneering work of Huggins and Hodges, which established PCa as a hormone-sensitive cancer, confirming its inhibited growth by the suppression of androgens levels or estrogen administration [6,7], has provided the foundations for the use of hormone therapy.

Estrogens were used in PCa treatment for several years, with the synthetic estrogen DES being a low-cost effective therapy for delaying the progression of metastatic PCa [7,8,9,10,11,12]. In the 1960s and 1970s, the Veterans Administration Cooperative Urological Research Group performed various randomized trials to evaluate the effectiveness of estrogenic therapies for PCa treatment, alone or in combination with orchiectomy [95]. This and other studies observed that estrogen therapy was able to delay the progression of PCa, accomplishing clinical responses in up to 80% of patients [8,9,10]. However, clinical trials also highlighted the adverse effects of hormone therapy, namely its hazardous effects at the cardiovascular level, with associated lethality [8]. A notorious increased risk of cardiovascular toxicity was found in up to 35% of patients receiving estrogen therapy, and thromboembolism was experienced by 15% [3,4,5].

The routes of estrogen administration are diverse (Table 2) and have evolved to accompany the monitoring of estrogens’ beneficial vs. adverse actions and minimize the extent of the harmful effects of hormones. Oral treatment is simple and convenient, and intestinal absorption is rapid [96]. However, in the intestine wall, about 70% of estradiol is metabolized into estrone, which only has one-third of the biological activity of estradiol [96]. In addition, the estrogens that reach the target organs are those that are not retained or excreted by the liver, and the concentration at these organs is about five times less than in the liver [97]. This required the use of doses that have been shown to produce toxicity, with relevant side effects such as hypertension, venous thromboembolism, pulmonary embolism, cerebrovascular accident, ischemic attack, and hypercoagulability, among others [98,99,100]. The parenteral route (intramuscular injection or subcutaneous injection) emerged to circumvent and alleviate the thromboembolic cascade of events and cardiovascular complications associated with oral administration by reducing the liver-associated toxicity and levels of several coagulation factors [101,102,103]. Nevertheless, parenteral treatment was linked to enhanced gynecomastia, intermittent claudication, and retinal thrombosis [104,105]. Several reports have demonstrated that transdermal patches (Table 2) have a similar efficacy compared with other administration routes with fewer side effects [106,107,108,109], which is interesting as they can be easily self-administered and readily withdrawn if toxicity occurs. Table 2 summarizes the information considering the administration routes and doses for different estrogenic formulations.

Despite the effectiveness in suppressing PCa growth and after decades of practice and attempts to optimize treatment approaches, estrogen therapy was withdrawn due to the associated adverse side effects.

Although the “estrogenic approach” was abandoned, research in the area has evolved over the years, disclosing the effects of estrogens and their mode of action [110].

**Table 2:** Estrogens in PCa treatment.

Route of Administration	Compound	Dosage	Ref.
Oral	Estradiol	1–2 mg 3×/day	[111]
	Conjugated estrogens	1.25–2.5 mg 3×/day	[112,113]
	Ethinylestradiol	0.15–3 mg/day	[112,113]
	Ethinylestradiol sulfonate	1–2 mg 1×/week	[114,115]
	DES	1–3 mg/day	[116]
	Dienestrol	5 mg/day	[117]
	Hexestrol	5 mg/day	[117]
	Fosfestrol	100–480 mg 1–3×/day	[118,119]
	Chlorotrianisene	12–48 mg/day	[120]
	Quadrosilan	900 mg/day	[117]
	Estramustine phosphate	140–1400 mg/day	[121]
Transdermal patch	Estradiol	2–6× 100 μg/day Scrotal: 1× 100 μg/day	[122,123]
Intramuscular or subcutaneous injection	Estradiol benzoate	1.66 mg 3×/week	
	Estradiol dipropionate	5 mg 1×/week	
	Estradiol valerate	10–40 mg 1×/1–2 weeks	[89,124]
	Estradiol undecylate	100 mg 1×/4 weeks	[125]
	Polyestradiol phosphate	Alone: 160–320 mg 1×/4 weeks With oral EE: 40–80 mg 1×/4 weeks	[126,127]
	Estrone	2–4 mg 2–3×/week	[91]
Intravenous injection	Fosfestrol	300–1200 mg 1–7×/week	[118,119]
	Estramustine phosphate	240–450 mg/day	[128]

### 3.2. Synthetic Estrogens

The effects of estrogens were, firstly, believed to be mostly mediated through the blockade of the hypothalamic–pituitary–gonadal axis, inhibiting gonadotropin-releasing hormone (GnRH) and luteinizing hormone (LH) release through negative feedback loops [129], resulting in decreased testosterone production and tumor regression [2,130]. This fact was supported by several reports in the 1990s that were not able to identify any detectable ER levels in the epithelial compartments of human prostatic tissue [131]. However, the paradigm changed with the characterization of ERs and the demonstration that estrogens have therapeutic effects through direct action in PCa cancer cells [132,133], independently of the systemic hypothalamic–pituitary–gonadal axis.

The human ERα cDNA was first cloned in 1985 [134,135], a discovery followed more than ten years later by the identification of a second *ER* gene, the *ERβ* [21]. ERβ is the most prevalent ER subtype expressed in human prostate tissue [136] and is mainly associated with the differentiation compartment that includes the luminal cells [137]. ERα expression in the non-neoplastic prostate is restricted to stromal cells and to the androgen-independent basal cell layer that comprises prostate stem cells and the proliferation compartment of the prostate epithelium [131,138,139].

The different expression pattern of the ER subtypes between luminal cells and basal cells modulates their susceptibility to cytotoxic agents [140]. Luminal cells expressing high levels of ERβ are particularly vulnerable and activate the programmed cell death after androgen deprivation therapy, radiation, or chemotherapy. In contrast, basal cells presenting high ERα expression levels are multi-drug resistant and survive to cytotoxic conditions [140]. Accordingly, the expression of ERβ in PCa was associated with a better relapse rate than ERβ-negative tumors [141], which renders this ER subtype a putative therapeutic target [142]. Regarding ERα, its expression was demonstrated to be upregulated during malignant progression of the prostatic epithelium and is highly expressed in metastatic PCa and CRPC [143]. Moreover, it has been shown that *ERα*-knockout mice showed no development of high-grade prostatic intraepithelial lesions or PCa upon chronic treatment with testosterone and estradiol [144]. Therefore, ERα inhibitors might have a potential antitumor activity in PCa. Overall, nuclear ER signaling seems to play a dual role in PCa development, progression, and therapy. GPER-mediated effects should also be taken in account, as this membrane receptor has been shown to trigger both tumor growth and tumor regression effects [18]. Moreover, it is crucial to fully characterize the ERs and GPER downstream signaling pathways and the effectors involved in the modulation of PCa progression [145].

**Figure 2:**
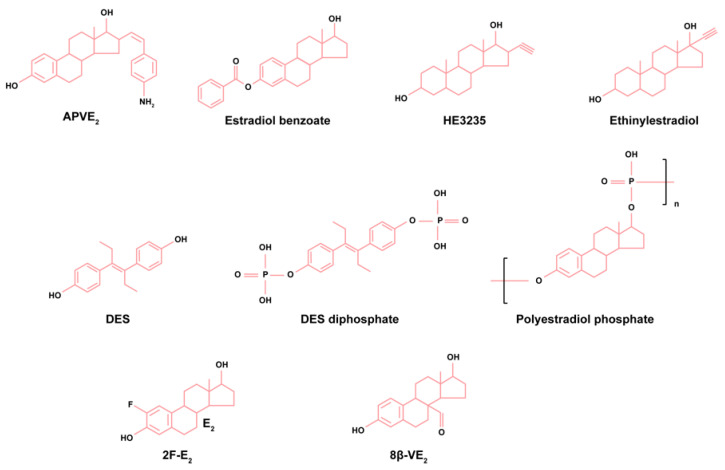
Structure of synthetic estrogens with reported effects in PCa. Legend: DES—diethylstilbestrol; HE3235—17α-ethynyl-5α-androstane-3α, 17β-diol; 8β-VE2—8β-vinylestra-1,3,5(10)-triene-3β,17β-diol; APVE_2_—17α-20Ζ-21-[(4-amino)phenyl]-19-norpregna-1,3,5(10),20-tetraene-3,17β-diolthe; 2F-E_2_—2-fluoroestradiol.

Considering the pharmacological interest and the ERs and GPER druggability, a large variety of synthetic estrogenic compounds with different structures and pharmacologic and metabolic characteristics have been investigated (Figure 2).

DES is a synthetic nonsteroid estrogen that, in contrast to the natural estrogen E_2_, is not markedly bound to sex hormone-binding globulin [146]. As described, it was the gold standard in endocrine therapy, though with significant side effects [147]. Ethinylestradiol is a synthetic and potent steroid estrogen since it suffers an alkylation in the 17α position, which does not allows it to be a substrate for 17β-hydroxysteroid-dehydrogenase, the enzyme that reversibly converts E_2_ to the less potent E_1_ (Figure 1) in target tissues [148,149].

In the attempt to discover better estrogen formulations to use in the treatment of PCa, novel compounds have emerged that include conjugated estrogens, polyestradiol phosphate, ethinylestradiol, the synthetic benzoate ester of estradiol (β-estradiol 3-benzoate), 17α-ethynyl-5α-androstane-3α, 17β-diol (HE3235), 8β-vinylestra-1,3,5(10)-triene-3β,17β-diol (8β-VE2), 17α-20Ζ-21-[(4-amino)phenyl]-19-norpregna-1,3,5(10),20-tetraene-3,17β-diolthe (APVE2), and the nongenotoxic estrogen 2-fluoroestradiol (2F-E2), among others (Figure 2). The detailed effects of these compounds in controlling prostate cell fate and tumor growth are presented in the subsequent sections of the review.

## 4. Estrogens as Prostate Carcinogens

### 4.1. Cell Survival and Neoplastic Transformation

Estrogens have been implicated as a cause of BPH, a condition predominantly characterized by the overgrowth of the stromal compartment. For example, E_2_ was shown to increase the proliferation of human BPH-derived stromal cells in an ER-dependent mechanism but had no effect on epithelial cells [150]. However, E_2_ (1 µM) was shown to increase the proliferation of immortalized nontumorigenic human prostate epithelial BPH-1 cells in culture for 3 days [151], although cell proliferation started being reduced after 6 days of hormone exposure [151]. Nevertheless, DNA damage was significantly higher in BPH-1 cells treated with E_2_ for 6 weeks, accompanied by an increased percentage of cells immunopositive for the oncogene *c-Myc*- and the cell cycle inducer cyclin D1 [151]. In recombinant prostate tissue, composed of urogenital mesenchyme plus BPH-1 cells grown under the kidney capsule of male athymic nude mice, testosterone and E_2_ treatment increased the Ki-67 proliferation index compared with the untreated animals [152].

Different types of studies, with diverse experimental approaches and using distinct estrogenic compounds, have demonstrated that estrogens can deregulate prostate cell survival and growth (Table 3), which have been linked to hyperplasia, dysplasia, metaplasia, and neoplasia.

The effects of estrogens inducing the proliferation of human prostate cells have been reported in different cell types, from non-neoplastic to androgen-sensitive and CRPC cells. E_2_ concentrations from 0.01 to 5 µM (Table 3) increased the proliferation of human prostate epithelial cells and androgen-sensitive LNCaP and VCaP cells [153,154,155]. In CRPC cells, namely, the DU145 and PC3 cell lines, E_2_ (0.01 and 1 µM) increased the anchorage-independent growth and inhibited apoptosis [14]. E_2_ also increased the growth and anchorage-independent growth of cells isolated from the lymph node metastasis of a PCa patient in an ERα-dependent mechanism [15].

The molecular orchestrators responsible for the proliferative actions of estrogens have also been disclosed. Besides the dependency of ERα alone, the interaction between ERα, androgen receptor (AR), and Src has been shown to be required for the rapid activation of the mitogen-activated protein kinase (MAPK) proliferation pathway in non-neoplastic cells [153]. Moreover, the augmented proliferation of LNCaP cells was associated with the increased expression of PSA, insulin-like growth factor 1 receptor (IGF-1R), IGF-1, insulin-like growth factor binding protein-3/4 (IGFBP-2), and the decreased expression of AR, ERβ, IGF-II, and IGFBP-3 [154].

The increased proliferation of prostate cell lines in response to estrogens has been translated to in vivo experiments. E_2_ administration (Table 3) was shown to decrease the population of rat prostate epithelial cells in phase G0/G1 while increasing the S and G2/M phase cell populations [13]. In addition, E_2_ caused DNA damage and induced apoptosis of prostate tissue cells, at least for short-term exposure [13].

For example, in human prostate xenografts, early exposure to estradiol benzoate (7 to 90 days) combined with secondary exposure (until 200 days) increased the proliferation of prostate epithelial cells, leading to the development of hyperplastic glands [156]. This was underpinned by the suppression of *PTEN* expression and likely augmented the activity of the PI3K-Akt signaling pathway, resulting in the inhibition of apoptosis and cell cycle progression [156]. Along the same line, DES has been reported as causative of prostate hyperplasia, since prenatal exposure to this compound resulted in enlarged prostates in adults [157]. Beyond the induction of cell proliferation, the effects of estrogens have been related to the development of other histological alterations in the prostate. Several studies have reported that E_2_, in combination with testosterone, induced dysplasia in rat dorsolateral prostate [158,159,160] as well as in the lateral, ventral, dorsal, and anterior regions of mice prostate [144].

Also, several estrogenic compounds seem to cause squamous metaplasia in the prostate of several species [161,162,163]. DES induced the metaplastic transformation of mouse prostate epithelium, mainly in the anterior region [164]. This effect was associated with the onset of cytokeratin-10 expression, the upregulation of progesterone receptor expression, and the loss of expression of the cell cycle inhibitor p27 ^Kip1^ [164].

Yu and colleagues observed that E_2_ induced the neoplastic transformation of rat prostate epithelial cells [13]. These transformed cells displayed an increased expression of several putative PCa stem cell markers as well as changes in the expression of hormone receptors, namely, increased levels of ERα and the decreased expression of ERβ and AR [13,151], which indicates a change in the hormone-responsiveness accompanying tumor development. Moreover, these findings support the driven role of ERα in prostate carcinogenesis. Low doses of E_2_ also increased tumor growth in DU145 and PC3 cell xenograft models [14]. However, the carcinogenic effects of estrogens have mainly been demonstrated by their association with testosterone (Table 3). Rat treatment with E_2_, in addition to testosterone, promoted tumor development, causing the formation of DNA adducts, oxidative DNA damage, and lipid peroxidation [160]. In mice, the pathologic areas induced by the testosterone plus E_2_ treatment showed an increased number of PCNA-positive proliferating cells [144]. Moreover, the addition of E_2_ was shown to shift the incidence of prostate tumors to 100% compared only to an incidence of 35–40% when testosterone was given alone [159]. These findings implicate estrogens in prostate carcinogenesis and indicate that the known effects of androgens driving cancer may depend on testosterone aromatization to E_2_, which has been demonstrated in animal models. Aromatase knockout mice, unable to synthesize E_2_, displayed a reduced incidence of PCa compared with wild-type animals in response to testosterone administration [144]. This study also showed that the presence of a functional ERα is decisive for tumor development. E_2_ plus testosterone treatment was ineffective, inducing PCa in *ERα* knockout mice, whereas *ERβ* knockout animals displayed a biochemical and histological pattern of carcinogenesis similar to their wild-type counterparts [144].

Humans are exposed to estrogens since embryological life, and prenatal exposure to estrogens has been shown to have observable effects in the adult male reproductive tract including the prostate gland [165]. Studies performed in animal models have shown that exposure to low doses of estrogens during fetal development causes the enlargement of the prostate [157]. In contrast, high doses of estrogens implicate a reduction in the adult prostate weight [157]. Moreover, developmental exposure to estradiol benzoate seems to change the differentiation and epigenetic programming in a human fetal prostate xenograft model [156], which can be a mechanism contributing to carcinogenesis later in life. Research efforts are needed to ascertain the impact of fetal estrogen exposure and the development of PCa in adulthood.

Catechol estrogens are potent compounds that originate from the metabolism of estrogens. This class of compounds has been identified in the prostate tissue and has also been linked to prostate malignancy [166]. Polymorphisms in the estrogen metabolism enzymes that detoxify catechol estrogens, namely, catechol-O-methyltransferase (COMT), glutathione (GSH), and quinone reductase are associated with PCa risk [167,168,169,170,171]. Furthermore, prostate areas susceptible to carcinoma development have been shown to have less protection through the activity of these enzymes. This susceptibility and the protumorigenic effects were related to the reaction of catechol estrogen-3,4-quinones (E_2_-3,4-Q) with DNA, which is favored by the reduced activity of COMT, GSH, and quinone reductase [166,172]. In vitro studies showed that exposure to the catechol estrogens 2-hydroxyestradiol (2-OHE_2_) and 4-hydroxyestradiol (4-OHE_2_) increased the proliferation of BPH-1 cells [151]. This was achieved by slightly diminishing the nonproliferating cell fraction (G0/G1-phase) while increasing the cell population in S-phase [151]. Moreover, both catechol estrogens increased the expression of cyclin D1 and c-Myc, which explains the shift toward proliferative behavior [151,173]. Increased proliferative ability driven by 4-OHE_2_ and 2-OHE_2_ was accompanied by the altered expression of molecular targets in estrogen signaling pathways. These catechol estrogens increased the abundance of ERα and its downstream target IGF-1R, whereas they reduced the levels of ERβ and its downstream tumor suppressor FOXO-1. However, 4-OHE_2_ effects were observed to a greater extent than that of 2-OHE_2_ [151]. Furthermore, genotoxic effects linked with the neoplastic transformation of BPH-1 cells were observed in response to the administration of 4-OHE_2_ and 2-OHE_2_ [151].

Catechol estrogens have also been shown to disturb tissue homeostasis and prostate histology architecture. The catechol estrogen 4-OH-E_2_, in combination with testosterone, induced prostatic dysplasia, and the frequency of appearance of this histological phenotype was doubled by the addition of 2F-E_2_ to testosterone and 4-OH-E_2_ [160].

In sum, several reports have defended that exposure to exogenous estrogens can be tumorigenic, mainly when low doses are used. The majority of in vitro studies have reported that E_2_, at least for concentrations below 1 µM (Table 3), increased the proliferation of prostate cells. Also noteworthy is the fact that the altered intraprostatic estrogen biosynthesis is associated with neoplastic transformation of the prostate by disrupting downstream signaling pathways and influencing the development and proliferation of prostate epithelial cells and stroma [174].

**Table 3:** Effect of estrogens in regulating the survival and growth of prostate cells in vitro and in vivo.

Type of Study	Cell Line/Animal Model	Compound	Concentration/Dose	Assay Model/Method of Administration	Time of Treatment	Assay	Effect	Activated Pathway	Ref.
In vitro	BPH-1	E_2_	1 µM	6-well plates	Up to 6 days/6 weeks	Sulforhodamine-B Comet	↑ Proliferation Neoplastic transformation	Genotoxic mechanism	[151]
	BPH-1	2-OHE_2_	1 µM	6-well plates	Up to 9 days/6 weeks	Sulforhodamine-B Comet	↑ Proliferation Neoplastic transformation	Genotoxic mechanism	[151]
	BPH-1	2-OHE_2_	10 µM	6-well plates	2, 4, 7 and 10 days	Sulforhodamine-B	↓ Cell viability	Cytotoxicity	[151]
	BPH-1	4-OHE_2_	1 µM	6-well plates	Up to 9 days/6 weeks	Sulforhodamine-B Comet	↑ Proliferation Neoplastic transformation	Genotoxic mechanism	[151]
	LNCaP	E_2_	0.01 µM	Multi-well plates	24 h	BrdU incorporation	↑ Proliferation	MAPK (ERK1/2) and association of AR, ERβ, and Src	[175]
	LNCaP	E_2_	0.01–5 µM	96-well plates	3 days	MTS PSA measurement	↑ PSA expression ↑ Cell growth	ER mediated	[176]
	LNCaP	αE_2_	0.01–5 µM	96-well plates	3 days	MTS PSA measurement	↑ PSA expression ↑ Cell growth	ER mediated	[176]
	LNCaP	E_2_	0.01 µM		24 h/48 h	WST-1 based	↑ Proliferation ↑ PSA expression	↑ c-Myc	[177]
	DU145	E_2_	0.01 µM	12-well plates	24 h	Soft-agar colony formation TUNEL	↑ Anchorage-independent growth ↓ Apoptosis	↓ FOXO1	[14]
	DU145	E_2_	1 µM	12-well plates	24 h	Soft-agar colony formation TUNEL	↑ Anchorage-independent growth ↓ Apoptosis	↓ FOXO1	[14]
	PC3	E_2_	0.01 µM	12-well plates	24 h	Soft-agar colony formation TUNEL	↑ Anchorage-independent growth ↓ Apoptosis	↓ FOXO1	[14]
	PC3	E_2_	1 µM	12-well plates	24 h	Soft-agar colony formation TUNEL	↑ Anchorage-independent growth ↓ Apoptosis	↓ FOXO1	[14]
	EPN	E_2_	0.01 µM	60 mm dishes	27 h/5 min	BrdU incorporation	↑ Proliferation	MAPK (ERK1/2) and association of AR, ERα, and Src	[153]
	NRP-152	E_2_	1 or 3 µM	96-well plates/ 60 mm dishes	2–6 weeks and 3–48 h	Soft agar colony formation Comet Flow cytometry	Neoplastic transformation	Genotoxic mechanism	[13]
	MDA-Pca 2b	βE_2_	0.01–5 µM	96-well plates	3 days	MTS PSA measurement	↑ Cell growth	ER mediated	[176]
In vivo	BALB/c mice	DES *	2 mg DES (and 18 mg cholesterol)	Pellet s.c. implant	Up to 3 weeks	PCNA-immunohistochemistry	↑ Proliferation	ERα	[164]
	Nude mice	E_2_	1 or 3 µM	E_2_-NRP-152 cells s.c. injection in the flanks	4–8 weeks	Tumor size measurement	↑ Neoplastic transformation	Genotoxic mechanism	[13]
	Athymic nude mice	β-estradiol 3-benzoate *	250 μg/kg (early exposure) + 2.5 mg pellet (secondary exposure)	S.c injection	90 days (early exposure) + 110 days (secondary exposure)	Ki-67 quantification	↑ Proliferation	PI3K-Akt pathway	[156]
	Xenograft BALB/ cA-nu castrated mice using DU145 and PC3 cells	E_2_	0.18 mg	Pellet s.c. implant	25 to 35 days	Tumor volume and weight measurement TUNEL	↑ Tumor growth	↓ FOXO1 ERβ and KLF5 pathway	[14]
	Athymic nude mice Tissue recombinants composed of mouse urogenital mesenchyme plus an immortalized nontumorigenic human prostatic epithelial cell line (BPH-1) grown under the kidney capsule	E_2_	2.5 or 10 mg (plus Testosterone)	Silastic implants	1–4 months	Immunohistochemistry Growth indices Determination of cancer incidence	↑ Proliferation ↑ Apoptosis	Akt pathway	[152]
	NBL/Cr rats	4-OHE_2_	5 μg/day	Silastic implants	13 weeks	Measurement of DNA adducts Measurement of 8-hydroxyguanosine Measurement of lipid hydroperoxides	↑ Inflammation ↑ Dysplasia	-	[160]
	NBL/Cr rats	2F-E_2_ *	5 μg/day	Silastic implants	13 weeks	Measurement of DNA adducts Measurement of 8-hydroxyguanosine Measurement of lipid hydroperoxides	↑ Inflammation ↑ Dysplasia	-	[160]
	NBL/Cr rats	E_2_ (+Testosterone)		S.c. silastic implants	91 weeks	Hematoxylin and eosin staining	↑ Prostate adenocarcinoma development	-	[159]
	NBL/Cr rats	DES * (+Testosterone)		S.c. silastic implants	91 weeks	Hematoxylin and eosin staining	↑ Prostate adenocarcinoma development	-	[159]
	Sprague- Dawley (Hsd:SD) rats	E_2_ (+Testosterone)		S.c. silastic implants	75 weeks	Hematoxylin and eosin staining	↑ Prostate adenocarcinoma development	-	[159]
	Sprague- Dawley (Hsd:SD) rats	DES * (+Testosterone)		S.c. silastic implants	75 weeks	Hematoxylin and eosin staining	↑ Prostate adenocarcinoma development	-	[159]
	CD-1 mice	E_2_ (+Testosterone)		Silastic implants	4 months	Histopathological grading Immunohistochemistry Histological analysis	↑ Prostate size	-	[144]
	C57BL/6 mice	E_2_ (+Testosterone)		Silastic implants	4 months	Histopathological grading Immunohistochemistry Histological analysis	↑ Prostate size Prostatic intraepithelial neoplastic lesions’ induction	↓ α-actin ↓ E-cadherin	[144]
	C57BL/ 6 X J129 mice	E_2_ (+Testosterone)		Silastic implants	4 months	Histopathological grading Immunohistochemistry Histological analysis	↑ Prostate size	-	[144]

* synthetic compound.

### 4.2. Progression of Disease and Metastization

Metastization is the complex process of cancer dissemination to distant organs and is the main cause of cancer-related deaths [178]. The occurrence of metastasis mainly depends on the migration and invasion capabilities of cancer cells, which is a consequence of the epithelial–mesenchymal transition (EMT) [179]. EMT is characterized by a switch in the expression of epithelial to mesenchymal markers, namely, the reduction in E-cadherin expression and the augmentation of N-cadherin and vimentin proteins [180,181], which in the case of prostate cells has been linked with estrogenic actions, and the effect of catechol estrogens.

Estrogens could alter the phenotype of BPH-1 cells, downregulating E-cadherin and upregulating vimentin and Snail expression, thus stimulating EMT [182]. Altered expression of EMT markers and enhanced migration and invasion properties have been described in PCa cells in response to estrogens. E_2_ was shown to promote the migration and invasion of distinct PCa cell line models and patient-derived cells (Table 4). E_2_ (0.01 µM) increased the migration of PacMetUT1 (isolated from the lymph node metastasis of a PCa patient) and 22Rv1 cells [15]. Accordingly, treatment with E_2_ caused a change in the morphology of PacMetUT1 cells similar to the EMT process, which was confirmed by a downregulation of E-cadherin expression and upregulation of vimentin and Snail [15]. Catechol estrogens, namely 2-OHE_2_ and 4-OHE_2_, have been shown to increase the invasion of prostatic cells [151], which can disrupt the prostate histology architecture and promote tumor progression.

In vivo approaches support the outcomes of cell-based research, indicating the role of estrogens in PCa progression and metastization (Table 4). Mice receiving E_2_ in combination with testosterone showed a dramatic reduction in α-actin and E-cadherin expression accompanying the prostate malignant transformation [144]. Similar findings were found when applying this treatment in mice harboring recombinant prostate tissue composed of urogenital mesenchyme and immortalized BPH-1 cells [152]. Metastatic carcinoma cells were identified in renal lymph nodes, lungs, and liver, with a drastic reduction in E-cadherin expression [152].

ERα has been indicated as the “guilty” ER in mediating the pro-migration and -invasion effects of estrogens. Indeed, the enhanced migratory capabilities observed in the PCa cell lines and patient’s metastasis-derived cells were reported to depend on ERα signaling [15]. However, ERα might promote the metastatic process by favoring the “seed” of PCa cells in other organs. The “seed and soil” hypothesis proposed by Stephen Paget to explain the emergence of metastasis [183] compares the dissemination of tumor cells to the distribution of seeds, arguing that only the seeds that find the proper soil will survive. The rationale underlying this model supports that cells from a primary tumor will establish metastasis if they find a well-matched organ. PCa preferentially metastasizes to bone [184], and interestingly, it has been shown that estrogen signaling through ERα stimulates the osteoblast-like properties of PCa cells, demonstrating its important role in the formation of osteoblastic lesions [15]. This suggests that estrogen signaling can be a relevant mechanism driving the progression of the disease for more aggressive stages and the emergence of bone metastasis. Therefore, characterizing the expression of ERs, mainly ERα, in PCa cases could be a useful prognosis tool in evaluating the progression of disease. Research is needed to deeply clarify this usefulness, though the presence of ERα, or the combination of ERα-positivity with low ERβ expression, is correlated with worse biochemical recurrence, disease progression, and survival outcomes [185].

The stroma component has also been shown to play a triggering role in the metastatic effects of estrogens. One interesting work revealed that conditioned medium from E_2_-stimulated prostate stromal cells could promote DU145 and PC3 PCa cell migration [186]. This increased migration seems to be mediated by enolase 1 [186], a key enzyme in glycolytic metabolism, also known as pyruvate dehydrogenase phosphatase, which catalyzes the transformation of 2-phosphate-D-glycerate to phosphoric acid-pyruvate as well as the reverse conversion of phosphoric acid-pyruvate to 2-phosphate-D-glycerate for glycogen synthesis [187]. Enolase 1 expression is frequently increased in tumors [188], and due to its role in anaerobic glycolysis, it is thought to promote tumor development and progression [187]. A total of E_2_ 0.01 µM enhanced the stability of enolase 1 in prostate stromal cells and promoted its secretion to the extracellular matrix in an ERα-dependent mechanism [186]. Studies with recombinant enolase 1 showed that this enzyme binds to the membrane of PCa cells, promoting cell migration in a paracrine manner via their plasminogen receptor activity [186]. The secretion of stromal cell-derived enolase 1 and its association with the surface of PCa cells recruits and activates plasminogen, thereby promoting the remodeling of the extracellular matrix and migration [186,189,190].

Another mechanism related to the effect of E_2_ promoting migration and invasion is the upregulation of the sex-determining region Y-box 4 (SOX4) [155], a developmental transcription factor overexpressed in many types of human tumors [191]. SOX4 was shown to regulate the expression of metastasis-related genes promoting EMT and PCa progression [192].

Furthermore, the augmented intraprostatic production of estrogens seems to promote the progression of CRPC and favor tumor metastasis. Aromatase knockdown and the aromatase inhibitor letrozole were shown to increase the overall survival and inhibit metastasis in castrated PC3 xenograft mice [193], which contributed to imply estrogens in PCa metastization. The estrogenic effects in the PC3 xenograft mice were proposed to occur through the activity of ERα, and its interaction with the estrogen response elements in the *MMP12* gene, regulating the expression of matrix metalloproteinase 12 (MMP-12) [193], a family member of a class of proteins (MMPs) involved in the remodeling of extracellular matrix, tumor progression, and metastization [194,195]. *MMP-12* knockdown suppressed the invasion of PC3 cells [193] and has been shown to mediate the interactions between PCa cells and bone marrow stromal cells during bone metastasis [196], which further supports the role of ERα as a driver of PCa metastization to the bone.

In summary, in agreement with the pro-proliferative effects, low concentrations/doses of estrogens support the increased migration and invasion of PCa cells, leading to tumor progression and mestastization.

**Table 4:** Effect of estrogens in promoting prostate cancer cell migration, invasion, tumor progression, and metastization.

Type of Study	Cell Line/Animal Model	Compound	Concentration/Dose	Assay Model/Method of Administration	Time of Treatment	Assay	Effect	Activated Pathway	Ref.
In vitro	LNCaP	E_2_	0.01 µM		24 h/72 h	Immunoblotting Wound healing Invasion	↑ Migration ↑ Invasion EMT	↑ N-cadherin ↑ Vimentin ↓ E-cadherin	[177]
	LNCaP	E_2_	0.01 µM	12-well plates	5 days	Alkaline phosphatase activity	↑ Osteoblast-like properties	ERα signaling	[15]
	LNCaP	E_2_	0.01 µM	96-well plates	72 h	Wound healing Invasion	↑ Migration ↑ Invasion	SOX4 up-regulation	[155]
	22Rv1	E_2_	0.01 µM	24-well Boyden chambers	18 h	Migration	↑ Migration	ERα signaling	[15]
	RWPE-1	E_2_	0.01 µM	96-well plates	48 h/72 h 8 days	RT-PCR analysis Western blot	EMT	ERα signaling	[182]
	C4–2	E_2_	0.01 µM	24-well Boyden chambers	18 h	Migration	↑ Migration	ERα signaling	[15]
	PacMetUT1 isolated from the lymph node metastasis	E_2_	0.01 µM	24-well Boyden chambers	18 h	Migration	↑ Migration EMT	ERα signaling	[15]
	DU145	Conditioned medium PrSC from BPH patients or WPMY-1 cells treated with E_2_	0.01 µM	15-cm dishes	48 h	Transwell migration Wound healing	↑ Migration	ERα signaling ENO1 effects via its plasminogen binding domain	[186]
	DU145	E_2_	0.01 µM		24 h	Wound healing Invasion	↑ Migration ↑ Invasion	ERα signaling ERβ signaling Galectin-3 signaling	[30]
	DU145	E_2_	0.01 µM		48 h	Invasion analysis Colony formation analysis (soft agar)	↑ Invasion	SRC	[197]
	PC3	E_2_	0.01 µM			Invasion analysis Colony formation analysis (soft agar)	↑ Invasion	SRC	[197]
	PC3	Conditioned medium PrSC from BPH patients or WPMY-1 cells treated with E_2_	0.01 µM	15-cm dishes	48 h	Transwell migration Wound healing	↑ Migration	ERα signaling ENO1 effects via its plasminogen binding domain	[186]
	PC3	E_2_	0.01 µM	Incubation of cells 24-well, 8.0-μm pore size	Overnight	Matrigel invasion	↑ Invasion	ERα/matrix metalloproteinase 12 axis activation	[193]
	PC3	E_2_	0.0001 and 0.01 µM		24 h	Wound healing Invasion Colony formation analysis (soft agar)	↑ Migration ↑ Invasion	ERα and ERβ signaling β-catenin signaling	[198]
In vivo	CD-1, C57BL/6, and C57BL/ 6 x J129 mice	E_2_	2.5 or 10 mg (+Testosterone)	Silastic implants	4 months	Histological analysis Immunohistochemistry	Carcinogenesis	ERα signaling	[144]
	NBL/Cr rats	E_2_		Silastic implants s.c.	16 weeks	Measurement of DNA adducts Measurement of 8-hydroxyguanosine Measurement of lipid hydroperoxides	↑ Tumor incidence	DNA adduct Oxidative DNA damage Lipid peroxidation	[160]
	NBL/Cr rats	4-OHE_2_	5 μg/day	Silastic implants	13 weeks	Measurement of DNA adducts Measurement of 8-hydroxyguanosine Measurement of lipid hydroperoxides	Carcinogenesis	-	[160]
	NBL/Cr rats	2F-E_2_ *	5 μg/day	Silastic implants	13 weeks	Measurement of DNA adducts Measurement of 8-hydroxyguanosine Measurement of lipid hydroperoxides	Carcinogenesis	-	[160]
	Athymic nude mice Tissue recombinants composed of mouse urogenital mesenchyme plus an immortalized nontumorigenic human prostatic epithelial cell line (BPH-1) grown under the kidney capsule	E_2_	2.5 or 10 mg (+Testosterone)	Silastic implants	1–4 months	Immunohistochemistry Growth indices Determination of cancer incidence	↑ Progression ↑ Metastization	Akt pathway	[152]

* synthetic compound.

## 5. Evidence of Estrogens as Protective Agents

### 5.1. Antiproliferative and Proapoptotic Effects

#### 5.1.1. Endogenous Estrogens

**Figure 3:**
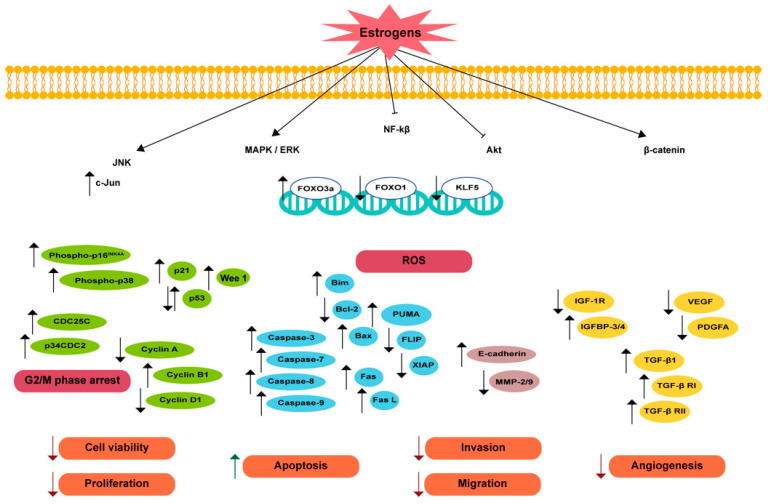
Antitumorigenic effects and signaling pathways activated by estrogens in controlling prostate cell survival, proliferation, apoptosis, invasion, migration, and angiogenesis. Summary of effects triggered by endogenous (E_2_, αE_2,_ 2-ME_2_, 3α-diol, 3β-Adiol) and synthetic (8β-VE_2_, APVE_2_, DES) estrogenic compounds according to the information provided in the text and Supplementary Table S1). JNK—c-Jun N-terminal kinase; MAPK—mitogen-activated protein kinase; ERK—extra-cellular signal regulated kinase; NF-κβ—factor nuclear kappa B; FOXO3a—Forkhead box class O 3a; FOXO1—Forkhead box protein O1; KLF5—Kruppel-like transcription factor 5; CDC25C—cell division cycle 25C; PUMA—p53 upregulated modulator of apoptosis; FLIP—FLICE-inhibitory protein; XIAP—X-linked inhibitor of apoptosis; Fas—Fas receptor; Fas L—Fas ligand; MMP-2/9—matrix metalloproteinase-2 and 9; IGF-1R—insulin-like growth factor 1 receptor; IGFBP-3/4—insulin-like growth factors binding protein-3/4; TGF-β1—transforming growth factor beta-1; TGF-β RI—transforming growth factor beta receptor 1; TGF-β RII—transforming growth factor beta receptor 2; VEGF—vascular endothelial growth factor; PDGFA—platelet derived growth factor subunit A.

In vitro and in vivo evidence have shown that E_2_, the most potent endogenous estrogen, is capable of reducing PCa cell viability and proliferation (Figure 3 and Supplementary Table S1). This effect was reported in PCa cell lines, either in androgen-sensitive, LAPC-4 and LNCaP as well as in 22Rv1 cells and AR-negative CRPC models, namely PC3 cells [147,176,199,200,201]. Treatment with E_2_ concentrations up to 10 µM was shown to reduce the DHT-induced cell growth of LNCaP and LAPC-4 cells [176,200], whereas lower concentrations, ranging from 1 nM to 1 µM, were able to diminish the proliferation of 22Rv1 and PC3 cell lines [199,201]. The same effect was shared by the E_2_ stereo-isomer 17α-estradiol (αE_2_), with 0.1–10 µM concentrations suppressing the proliferation of the androgen-sensitive PCa cell lines [176,200]. Moreover, the E_2_ effects counteracting the proliferation and viability of PCa cells were followed by an increase in apoptosis. E_2_ concentrations between 1 nM and 1 µM increased apoptosis in the LAPC-4 cells and also in the CRPC cells, namely, 22Rv1 and PC3 [147,199,201]. In addition, the stimulation of PCa cells with both E_2_ and αE_2_ had other interesting effects such as reversion of the DHT- or cortisol-induced PSA expression and a reduction in the DHT-induced DNA biosynthesis [176,200].

The results obtained in vitro were translated into the in vivo setting. Wistar rats treated with a supraphysiological dose of E_2_ showed a marked reduction in prostate weight underpinned by decreased cell proliferation and increased apoptosis compared to rats receiving a placebo [202]. Several studies in animal tumor models (Supplementary Table S1) have also reported the effects of estrogens counteracting the growth of malignant prostate tissue (Figure 3). In rodents, E_2_, αE_2_, or estradiol benzoate treatment reduced the prostate tumor growth (size and volume) by suppressing the proliferation of tumor cells and inducing apoptosis in both androgen-sensitive and CRPC tumor models [14,133,176,200,203,204,205,206]. In addition, survival improvement was described in the mice LuCaP 35 xenograft tumor model treated with E_2_ [133]. Also noteworthy are other reported effects of E_2_ in mice including the reduction in testosterone and DHT serum concentrations and PSA levels [133,200], which further corroborates the antitumorigenic effects of this hormone.

Additionally, the antitumor role of the endogenous metabolite of E_2_, 2-methoxyestradiol (2-ME_2_), has been widely studied in PCa. Kumar et al. [207] were the first to demonstrate that 2-ME_2_ inhibits the growth of non-neoplastic prostate epithelial cells. This growth inhibitory effect and the reduction in cell proliferation by 2-ME_2_ were also observed in a variety of both androgen-sensitive and CRPC cell line models (Figure 3 and Supplementary Table S1) [207,208,209,210,211,212]. Unsurprisingly, 2-ME_2_ has been highly associated with apoptosis induction in PCa (Supplementary Table S1) [207,208,209,210,211,213,214,215,216,217,218].

In vivo findings in rats and mice further supported the enormous quantity of in vitro evidence of the antitumorigenic role of 2-ME_2_. Copenhagen X Fisher F1 male rats transplanted with Dunning R3327-PAP prostate tumors treated with 12.5 mg/kg/day of 2-ME_2_ showed reduced tumor growth accompanied by increased apoptosis [213]. Accordingly, in both androgen-sensitive or CRPC xenograft mice models, 2-ME_2_ treatment could reduce tumor growth and augment apoptosis (Supplementary Table S1) [208,210,211,218,219,220,221]. Additionally, in the TRAMP mice model, in which tumor development resembles the progression of human PCa (hyperplasia, 8–12 weeks; neoplasia, 15–18 weeks; metastasis, 24 weeks), the effect of 2-ME_2_ treatment in reducing tumor growth was confirmed. The administration of 2-ME_2_ had an important role during the various stages of PCa, reducing prostate weight, malignant transformation, and neoplastic progression as well as promoting tumor regression [210,218,221]. Moreover, these effects were accompanied by a reduction in testosterone [221] and PSA levels [210]. However, Ganapathy and colleagues [218] observed a reduction in apoptosis in TRAMP mice treated with 2-ME_2_, which was suggested to be related to restoring normal tissue architecture.

Some years ago, it was demonstrated that the DHT metabolites, 5α-androstane-3α,17β-diol (3α-diol) and 5α-androstane-3β,17β-diol (3β-Adiol), have estrogenic activities via the activation of ERβ [222,223,224]. Also, these estrogenic metabolites of DHT were shown to have anticancer properties. A total of 0.1 µM (and concentrations below) 3β-Adiol diminished proliferation and increased the apoptosis of both androgen-sensitive and CRPC cells [225,226,227]. Accordingly, the proliferation of established tumors in PC3 xenograft mice tumor models was substantially reduced by a 3-week treatment with 3β-Adiol [226].

ERs and GPER as well as their downstream effectors in signaling pathways are the molecular players involved in the control of prostate cell growth by estrogens [147,176,200]. However, the protective role of estrogens in PCa has mainly been attributed to ERβ, which has been shown to have antiproliferative and proapoptotic effects in PCa models [228,229].

Over the years, the downstream effectors of the ERs’ actions have been disclosed. The stem cell factor (SCF) and its tyrosine kinase receptor c-KIT, with a recognized role in controlling cell proliferation and apoptosis, are important drivers of carcinogenesis in several tissues [230,231,232]. The SCF/c-KIT system was shown to be involved in non-neoplastic prostate cell growth and a target of estrogen regulation. Treatment with supraphysiological doses of E_2_ strongly diminished the rat prostate weight, suppressed cell proliferation, and induced apoptosis, underpinned by the diminished expression of SCF and c-KIT [202].

In PCa cells and tissues, the actions of estrogens in controlling cell proliferation strictly depend on the control of several cell cycle regulators, namely, cyclins, cyclin-dependent kinases (CDKs), and CDK inhibitors. The antiproliferative effects of estrogenic compounds such as αE_2_, E_2_, and 2-ME_2_ have been related to cell cycle arrest at the S, G1, and G2/M phases. αE_2_ and E_2_ seem to reduce cyclin A and cyclin D1 expression [200] and are involved in cyclin D1 phosphorylation through p38 kinase activation [147]. The reduction in these cyclins disrupts cell cycle progression, decelerating PCa proliferation. 2-ME_2_ was shown to induce G2/M arrest, increasing the G2/M population, with a concomitant decrease in the G1 population [207]. This G2 block can be explained by the inhibition of CDK-1 activity, since 2-ME_2_ leads to the accumulation of phosphorylated-CDK-1 by inducing the expression of the inhibitory Wee 1 kinase, which mediates the phosphorylation of CDK-1 at the tyrosine 15 residue, making it inactive [207,233,234]. 2-ME_2_ administration was also shown to increase the expression levels of p53 and CDK inhibitor p21, which further detailed the molecular targets underlying the effect of this estrogenic compound disrupting the cell cycle (Figure 3) [211,235].

Concerning the mechanisms involved in the proapoptotic effects of estrogens, both the extrinsic and intrinsic pathways of apoptosis are activated by E_2_ and estrogenic metabolites (Figure 3). E_2_- and 2-ME_2_-induced PCa cell apoptosis was shown to be mediated by the membrane death receptor Fas and its ligand (Fas L), with the subsequent activation of caspase-8 [202,215,218]. In fact, E_2_ and its metabolite 2-ME_2_ increased the expression of Fas and Fas L as well as caspase-8 [202]. Activation of the extrinsic pathway of apoptosis upon Fas/Fas L binding causes the formation of the death inducing signaling complex (DISC) by the recruitment of several molecules including the Fas-associated death domain (FADD) and procaspase-8, which becomes activated [236,237,238]. 2-ME_2_ was shown to augment DISC formation by inhibiting Akt, sensitizing PCa cells to Fas-mediated apoptosis [215]. The antiapoptotic protein Fas-associated death domain interleukin-1 converting enzyme-like inhibitory protein (FLIP) is a key inhibitor of Fas-induced apoptosis by competing with procaspase-8 for FADD binding, thus preventing the recruitment of caspase-8 to FADD, or by inhibiting caspase-8 activation through DISC [239,240]. Several studies have reported that PCa cells treated with 2-ME_2_ displayed diminished FLICE-inhibitory protein (FLIP) expression (Figure 3) [214,215,218].

2-ME_2_ proapoptotic effects through the activation of the intrinsic pathway of apoptosis encompass the regulation of several members of the Bcl-2 family, altering the ratio of and antiapoptotic proteins. This seems to occur due to the indirect effect of 2-ME_2_ reducing the levels of phosphorylated Akt as well as by other mechanisms [213,217,220,241,242,243,244,245,246]. Exposure to 2-ME_2_ increased the levels of the proapoptotic proteins Bax and Bim and reduced the expression of Bcl-2, an antiapoptotic member of the family [213,218,220,247]. The proapoptotic Bid, which promotes the leakage of cytochrome C from mitochondria, connects the intrinsic and extrinsic apoptotic pathways as it is activated by caspase-8 [248,249]. A study showed that 2-ME_2_ could induce Bid cleavage with consequent reduction in Bcl-2 and caspase-8 activation [218].

The 2-ME_2_ induction of apoptosis in PCa cells also seems to be correlated with the reduced levels of X-linked inhibitor of apoptosis protein (XIAP), a member of the inhibitor of apoptosis family of proteins (IAP) that inhibits caspase activity [235].

The proapoptotic actions of the 3β-Adiol metabolite were shown to induce the apoptosis of PCa cells through the intrinsic pathway with a reduction in Bcl-2, increase in Bax expression, and involvement of caspase-9, which culminates in the augmented activity of caspases-3/7 [225]. Furthermore, the 3β-Adiol activation of apoptosis by the mitochondrial pathway seems to be triggered by the increased transcription of the transcription factor Forkhead box class O 3a (FOXO3a), which causes an increase in the expression of p53 upregulated modulator of apoptosis (PUMA) (Figure 3) [225].

#### 5.1.2. Synthetic Estrogens

Several synthetic compounds with estrogenic activity have been developed over the years with the perspective of being used as anticancer molecules, suppressing PCa cell growth. With this rationale, the synthesized compounds were generally developed as ERβ agonists, as this receptor has been assigned to have antiproliferative and proapoptotic properties. Examples include the 8β-VE_2_ and APVE_2_ compounds (Figure 3 and Supplementary Table S1).

High doses of 8β-VE_2_ induced apoptosis in the PCa cell lines and the prostate of rodent tumor models (Supplementary Table S1), which was related to the interference in androgen/AR signaling. Androgens are the main players promoting PCa cell survival, and androgen deprivation is well-known to block prostate cell growth [250,251]. For this reason, androgen deprivation therapy (ADT) is a gold standard in PCa treatment. A recent study demonstrated that stimulating ERβ activity with the specific agonist 8β-VE_2_ diminished survival and increased the apoptosis of VCaP cells [252]. This human PCa cell line overexpresses the *AR* and its splice variants are linked with the resistance to ADT. Interestingly, 8β-VE_2_ treatment reduced the overexpression of *AR* and *AR* variants in VCaP cells under androgen deprivation [252], which renders this compound interesting in overcoming resistance to treatments. Results in mice models of BPH and PCa treated with 8β-VE_2_ demonstrated that ERβ causes apoptosis through the activation of the extrinsic pathway and TNFα mediation in an androgen-independent manner [253,254]. Other studies have reported that 8β-VE_2_ induces the apoptosis of PCa cells by activating both the extrinsic and intrinsic pathways [225,253]. 8β-VE_2_ has been shown to increase the expression of caspase-8, with the subsequent activation of caspase-3, in a TNFα mediated process [253]. Concerning the intrinsic pathway, 8β-VE_2_ reduced Bcl-2 expression and increased the cleavage of caspase-9 [225]. Furthermore, 8β-VE_2_-induced apoptosis by the intrinsic pathway seems to be mediated by the increased levels of FOXO3a [225]. The APVE_2_ compound was shown to reduce PCa cell growth by arresting the cell cycle at the G2/M phase and inducing apoptosis, with effects perceived at nanomolar concentrations (Supplementary Table S1) [255]. Studies concerning the antiproliferative and proapoptotic actions of APVE_2_ are scarce, and the mechanism is highly unknown.

Another well-known synthetic estrogen is DES, which has been widely used for CRPC treatment [98]. This compound diminishes PCa cell viability [256], reduces proliferation, and augments apoptosis (Figure 3) [147,257,258]. DES treatment depleted the population of CRPC cells in the G1 and S-phase, with an accumulation of cells in the G2/M phase [257,259]. DES has been shown to decrease the phospho-ERK levels and c-Jun N-terminal kinase (JNK) activation, increase the phosphorylation of the cell-cycle inhibitor protein p16^INK4A^, and activate p38 kinase, which then phosphorylates cyclin D1, tagging it for degradation and arresting cell cycle. This process was shown to be mediated by ERβ as well as by GPER [147].

Furthermore, the actions of DES suppressing cell division include the inhibition of telomerase activity, which is highly upregulated in dividing cells such as malignant cells [258] and the disruption of microtubule organization [259]. PCa cells stimulated with DES showed a decreased density of microtubules [259], with the suppression of β-tubulin isotypes I, II, and IV, and α-tubulin isotypes I and IV as well as the diminished expression of glyceraldehyde-3-phosphate dehydrogenase [259], which is a microtubule-associated protein that plays a role in microtubule bundling [260].

In addition, DES has been shown to inhibit AR transactivation activity in the PC3 and DU145 PCa cell lines, which indicates its antiandrogenic effects by attenuating the levels of AR-mediated transcription [261]. Other mechanisms associated with DES include the inhibition of HIF1α activation and changes in a panoply of metabolites including lactate, phosphocreatine, and reduced glutathione [262]. This suggests that DES can have a putative role in the metabolic rewiring of PCa cells.

HE3235 is another synthetic estrogen, which was suggested as potential clinical use for treatment of CRPC. HE3235 significantly decelerated tumor growth, decreased AR expression, lowered intratumoral testosterone and DHT levels, and inhibited the development of bone tumors [263,264]. 

Not surprisingly, the review of the existing knowledge concerning the role of estrogens in controlling PCa growth showed the consistent and powerful effects of these hormones in counteracting PCa cell proliferation (Figure 3). Indeed, these beneficial effects have been known for decades, with estrogens being used in PCa therapy, and only disregarded because of the important adverse effects reported. Notwithstanding, the evidence of the antitumorigenic role of estrogens has prompted the appearance of studies evaluating the effect of these hormones in combination with both chemotherapeutic drugs and natural compounds [201,211,212,220,265] as a strategy that could potentiate the action of each compound alone, allowing for a reduction in the doses used, and therefore, overcoming the problems related with collateral effects.

### 5.2. Suppression of Metastization

Tumor progression is a complex process encompassing several biological mechanisms that determine the molecular, morphological, and functional changes in cancer cells and tumor microenvironment. These changes result in the reorganization of the extracellular matrix, the promotion of angiogenesis, inflammation, and modulation of the immune system [266], which altogether drive tumor progression, invasion, and metastization.

Loss of cell–cell adhesion, which depends on the disruption of cell-to-cell and cell–matrix interactions, with the involvement of several molecular players, is critical for the occurrence of cell migration and invasion. E_2_ and 2-ME_2_ seem to interfere with tumor progression by upregulating and stabilizing the β-catenin protein, an important molecule in cell–cell adhesion [217,247,267]. β-catenin is a key component of the cadherin/catenin complex that mediates calcium-dependent homophilic interactions between cell adhesion molecules [268]. Furthermore, 2-ME_2_ has been shown to reduce the migratory ability of PCa cells by increasing the interaction of β-catenin with E-cadherin [247]. E-cadherin is the most abundant adhesion molecule in epithelial adherens junctions, and its loss is frequently associated with migration, invasion, and metastatic dissemination [269]. The estrogenic metabolite 3β-Adiol increased E-cadherin expression and cell adhesion, which resulted in a reduction in migration and invasion [226,227]. In vivo results confirmed that 3β-Adiol decreased PCa progression with the ability to reduce metastasis formation [226]. Furthermore, it was reported that 3β-Adiol controls the progression of PCa through the activation of ERβ [226,227]. Accordingly, studies in CRPC cells reported that ERβ activation leads to the increase in E-cadherin and β-catenin expression and the reduced expression of N-cadherin [267,270].

Considering MMPs, other important molecules involved in tumor cell invasion and metastasis, Kanagaraj et al. [199] reported that E_2_ was able to reduce the expression levels of MMP-2 and MMP-9. These findings are highly interesting as both MMPs are prostate gland secretion products, and their higher levels are related to the aggressive behavior of PCa and metastization [271,272].

The angiogenic process is essential for tumor cell survival, growth and metastasis by providing nutrients and oxygen to the growing tumor cells [273]. Angiogenesis comprises endothelial cell proliferation and migration, which can be activated by tumor cells via paracrine factors or direct cell-to-cell communication [274,275]. The vascular endothelial growth factor (VEGF) is a crucial angiogenic factor with mitogenic and antiapoptotic effects. It regulates angiogenesis by inducing the proliferation, migration, and permeability of endothelial cells [276]. VEGF expression is highly enhanced in developing tumors [277]. LAPC-4 or LNCaP cells in conditioned media were shown to induce the growth of a murine endothelial cell line (MEC), and VEGF was the main factor responsible for this paracrine stimulation [278]. E_2_ administration inhibited the paracrine effect of DHT in stimulating MEC proliferation [278]. Accordingly, both E_2_ and its stereoisomer αE_2_ reduced the number of microvessels in the prostate tumor tissues of the LAPC-4 or LNCaP xenograft models [278]. Additionally, E_2_ seems to suppress angiogenesis by inhibiting the ERβ- and KLF5-mediated expression of platelet-derived growth factor subunit A (PDGFA) [14]. This factor plays an essential role in regulating cell migration and chemotaxis [279], and its overactivity can trigger carcinogenesis [280]. The E_2_ metabolite 2-ME_2_ has also displayed antiangiogenic properties. 2-ME_2_ administration to LNCaP and PC3 xenografts reduced the prostate microvessel density [211,219,220], which occurred by decreasing the VEGF expression [220].

Estrogenic compounds have also been shown to control inflammation in PCa. This is the case of 2-ME_2_, which inhibited the progression of PCa in TRAMP mice by increasing the expression of TNF-α-stimulated gene 6 (TSG-6), a secreted glycoprotein with anti-inflammatory action [221]. No further evidence exists of the anti-inflammatory actions of estrogens either in vitro or in vivo. Nevertheless, a study in noncancer patients showed that circulating estrogens were inversely associated with intraprostatic inflammation [56], which suggests that these hormones can have a role in controlling prostate inflammation and PCa progression.

## 6. The Phytoestrogen Scope

Among all the natural products that have been used in improving human health as either preventive supplements or treatments, phytoestrogens are a class of compounds of particular interest. The relevance of phytoestrogens in human health relies on their wide distribution in several types of plants and fruits and their potent and diverse biological effects. Phytoestrogens have recognized antioxidant, antiproliferative, antiangiogenic, and proapoptotic activity [36], making them highly attractive for exploitation in the context of anticancer therapy. Furthermore, many epidemiological studies support the protective role of phytoestrogens in malignancy. For example, the incidence of breast and PCa is much lower in Asian people when compared to Westerners [281]. The average daily consumption of phytoestrogens in Asia is estimated to range from 20 to 50 mg, whereas in the United States and Europe, it is predicted to be 0.153 mg and 0.491 mg, respectively [282]. This astonishing difference is justified by the dietary habits of the Asian population, with a plant-based diet and much less consumption of meat and animal-derivative products [283], and it has been postulated to explain the lower incidence and mortality rates of PCa in Eastern countries [284,285].

### 6.1. Classification and Structure

Phytoestrogens are a group of natural biologically active compounds derived from plants and fruits, which, because of their analogous structure to the principal estrogen, E_2_, display estrogenic and/or antiestrogenic activity [286]. A great group of phytoestrogens are the phenolic compounds, encompassing two main classes of molecules: flavonoids and nonflavonoids. The first group includes flavones, isoflavones, flavanones, flavonols, and coumestans [287,288,289]. Stilbenoids (and lignans) are included in the nonflavonoid class [287,288]. Supplementary Table S2 lists the types of phytoestrogens with an identified role in modifying human prostate cell fate.

### 6.2. Sources and Metabolism

Phytoestrogens are abundant in nature and are mainly present in the fruits, vegetables, and whole grains usually consumed by humans [290]. Moreover, they are also found in several edible and/or medicinal plants [290]. Plant extracts with recognized estrogen-like activity comprise soy, red clover, kudzu, hops, licorice, rhubarb, yam, and chasteberry, among others [291].

After consumption, phytoestrogens are metabolized by intestinal bacteria, absorbed, conjugated in the liver, disseminated in plasma, and expelled in urine [292]. Gut metabolism has been shown to play a crucial role in determining the effectiveness of the actions of phytoestrogens. For some members of this class of compounds, the estrogenic effect is mainly due to the metabolites generated by digestion, rather than by the original phytoestrogen molecule [293]. For example, daidzein is a phytoestrogen that is converted to a more active metabolite, equol, with enhanced estrogenic activity compared with the original molecule [294]. However, the effect of equol is variable, depending on individual specificities since the ability for this conversion and the extension of this reaction appears to be restricted to approximately a third of the population [294]. Moreover, the bioavailability and uptake of some isoflavones require some metabolic processes that allow absorption into the peripheral circulation such as hydrolysis and reconjugation to glucuronic and sulfuric acids [295,296].

Despite some limitations on bioavailability and bioaccessibility, the potential of phytoestrogens as anticancer molecules is unquestionable, and continuous research would help in identifying new preventive or treatment approaches.

### 6.3. Mechanisms of Action

As above-mentioned, the molecular structure of phytoestrogens is very similar to E_2_, and they can bind ERα and ERβ. However, the binding affinity seems to be higher for ERβ [297,298]. After ligand binding, the receptors are translocated to the nucleus, where they bind to specific regulatory regions, the estrogens’ response elements, modulating gene transcription, and consequently, the cell protein network and activity [299]. Besides their ability to bind to ERs, phytoestrogens can modulate the activity of other transcription factors and pathways, namely serotoninergic and IGF-1 receptor signaling [300,301,302]. The effect of these compounds can also be exerted by binding free radicals, inducing DNA methylation and histone modification and modulating tyrosine kinase, cAMP/protein kinase A, cGMP/NO, PI3K/Akt, and MAP kinase activity [303,304,305,306].

In addition to classical ERs, phytoestrogens have also been shown to activate GPER [307].

### 6.4. Phytoestrogens Actions against Prostate Cancer

#### 6.4.1. Apigenin

Apigenin, also known as 4′,5,7,-trihydroxyflavone, is a nontoxic and nonmutagenic flavonoid widely present in common fruits and vegetables [308]. This phytoestrogen has been proven to have anti-inflammatory and anticarcinogenic effects in preclinical studies. Several types of cancer are sensitive to apigenin, namely leukemia, breast, colon, lungs, skin, thyroid, and prostate [309,310,311].

**Figure 4:**
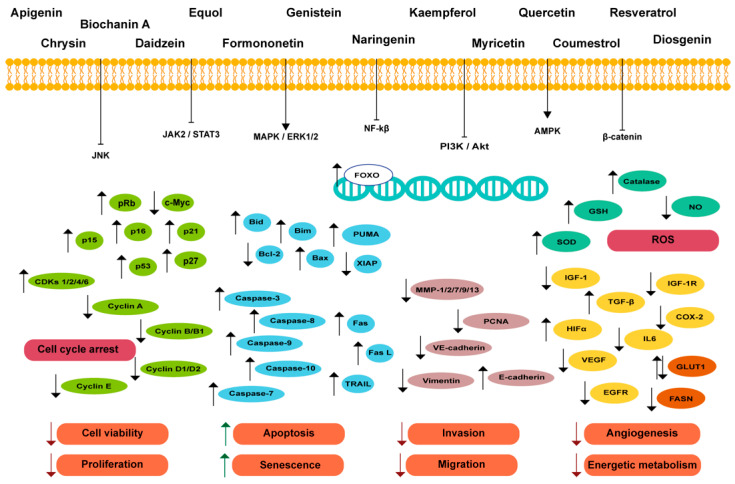
Phytoestrogens with a role in controlling PCa cell survival, proliferation, apoptosis, invasion, migration, and angiogenesis and activated signaling pathways. JNK—c-Jun N-terminal kinase; JAK2—Janus kinase 2; STAT3—signal transducer and activator of transcription; MAPK—mitogen-activated protein kinase; ERK—extra-cellular signal regulated kinase; NF-κβ—factor nuclear kappa B; PI3K—phosphatidylinositol 3′-kinase; AMPK—AMP-activated protein kinase; FOXO—Forkhead box; pRb—retinoblastoma protein; CDKs 1/2/4/6—cyclin-dependent kinases 1/2/4/6; PUMA—p53 upregulated modulator of apoptosis; XIAP—X-linked inhibitor of apoptosis; Fas—Fas receptor; Fas L—Fas ligand; TRAIL—TNF-related apoptosis-inducing ligand; MMP-1/2/7/9/13—matrix metalloproteinase 1/2/7/9/13; IGF-1—insulin-like growth factor 1; IGF-1R—insulin-like growth factor 1 receptor; TGF-β—transforming growth factor beta; HIFα—hypoxia-inducible factor α; COX-2—cyclooxygenase-2; IL6—interleukin 6; VEGF—vascular endothelial growth factor; EGFR—epidermal growth factor receptor; GLUT1—glucose transporter type 1; FASN—fatty acid synthase.

The effects of apigenin have been shown to inhibit PCa growth, reducing cell proliferation and inducing cell cycle arrest at the G2-M phase (Figure 4) [312,313,314,315]. In LNCaP and DU145 cells, cell cycle inhibition by apigenin has been linked to a marked decrease in the expression of cyclins D1, D2, and E and their activating partners CDK2, 4, and 6 [316,317]. This phytoestrogen also increased the expression of tumor suppressor protein p53 and cell cycle inhibitor p21 [313,316,317,318,319,320,321] and caused the dephosphorylation and inactivation of retinoblastoma protein (Rb) (Figure 4) [322].

The antiproliferative effect of apigenin seems to be mediated through the activation of ERβ or the suppression of the IGF axis, one of the well-known crucial pathways for cell proliferation [323,324,325]. Although no studies were performed on PCa, apigenin also acts through GPER [326].

Apigenin’s actions are also significant in relation to the control of NF-κB [327,328] and MAPK and Akt/PI3K survival pathways [322,329]. Inactivation of the Akt pathway, and stimulation of the production of reactive oxygen species (ROS), culminated in the apoptosis of PCa cells [321,330,331]. Akt is known to phosphorylate the Bad protein, which renders the antiapoptotic Bcl-2 protein free and activated and cells resistant to death. Thus, Akt inactivation is promptly linked to increased apoptosis as Bcl-2 is inactivated. Apigenin treatment has been shown to shift the Bax/Bcl-2 ratio toward apoptosis by increasing Bax and reducing Bcl-2 expression (Figure 4) [316,318,320,321].

Existent reports also indicate that apigenin can induce apoptotic cell death triggered at the cell membrane. Stimulation of PCa cells with apigenin strongly induced the expression of DR5 and TNF-related apoptosis-inducing ligand (TRAIL) death receptors [332,333] as well as caspase-8, caspase-10, caspase-9, and caspase-3 [317,330,331,332].

Of note, apigenin treatment significantly decreased the viability of cancer cells and augmented apoptosis, in contrast to the low magnitude of effects observed in non-neoplastic cells [312,332], indicating a selectivity over PCa cells.

Apigenin also inhibited the cellular processes related to cancer progression such as EMT, migration, and invasion [334]. Exposure to apigenin resulted in decreased cell motility and the reversion of EMT [334,335] as well as the blockade of β-catenin signaling [336] and inhibition of the migration and invasive potential of PCa cells [337]. These effects were shown to be dose- and time-dependent [337].

In LNCaP and PC3 cells, apigenin treatment also inhibited the expression of VEGF and hypoxia-inducible factor α (HIFα), angiogenesis, and hypoxia markers, respectively [338,339,340]. HIFα is a known driver of the hyperglycolytic phenotype of cancer cells in response to hypoxia by regulating the expression of several targets in the glycolytic flux, namely, glucose transporters (GLUTs), as in the case of GLUT1 [341]. Accordingly, in the presence of apigenin, PCa cells displayed a decreased expression of HIFα, accompanied by the diminution of GLUT expression [342,343], which likely limited the nutrient availability, contributing to suppress proliferative activity. Exposure of PCa cells to apigenin also diminished the activity of fatty acid synthase (FASN) [342,344], a target metabolic regulator whose expression is highly increased in PCa [345]. Overall, these findings indicate the potential of apigenin to counteract the metabolic rewiring in PCa.

The beneficial properties of apigenin could be observed in animal models [346], and one study reported that a herbal extract containing apigenin was able to enhance the chemotherapeutic effect of docetaxel in PCa [347]. Furthermore, apigenin combined therapy enhanced the efficacy of abiraterone acetate [348] and doxorubicin [349], and sensitized the PCa cells to radiation [350]. Moreover, apigenin sensitized PCa stem cells to therapy, which is relevant as this cell population is generally resistant to therapy and is believed to be involved in the nonresponse of PCa to anticancer drugs and disease recurrence [37,38]. Overall, the present data support the interest in this natural agent.

#### 6.4.2. Chrysin

Chrysin, also known as 5,7-dihydroxyflavone, is a flavone that can be extracted from honey, propolis, and blue passion flowers [351]. This agent is considered as a valuable resource for health purposes as it shows anticancer, antiangiogenenic, anti-inflammatory, antidiabetic, antibacterial, antiaging, and antiallergic properties [352,353,354,355,356,357,358]. Chrysin exhibited some binding activity to the nuclear ERs and GPER [359,360,361]; however, there is no evidence of the ERβ- or GPER-mediated actions of this compound in PCa. Nevertheless, chrysin suppressed proliferation and induced the apoptosis of PC3 cells [362,363,364]. One of the mechanisms by which this substance induces apoptosis is through the production of ROS [363]. HIF-1α is another signaling pathway that seems to have a role in chrysin anticancer activity [365]. In DU145 cells, chrysin decreased HIFα expression by inhibiting its protein synthesis (Figure 4) [352].

In parallel to the effects controlling PCa cell fate, a report showed that the use of chrysin as a complement to docetaxel increased the therapeutic efficacy of this anticancer drug, mitigating some side effects such as edema [366].

#### 6.4.3. Biochanin A

Biochanin A, 5,7-dihydroxy-4′-methoxyisoflavone, is an *O*-methylated isoflavone that can be found, for example, in red clover, soy, alfalfa sprouts, peanuts, and other legumes [367]. This flavonoid has been shown to induce a dose-dependent inhibition of LNCaP, DU145, and PC3 cell proliferation and viability [319,368]. Furthermore, it has been suggested that ERβ could mediate the antiproliferative effect of biochanin A, as this compound stimulated the receptor expression in PC3 cells [368]. Although biochanin A action through GPER has been reported [369], no studies have been caried out in PCa.

The capability of biochanin A to suppress cell division, with the accumulation of PCa cells in the G0 phase [370], was related to the decreased expression of cyclins B and E and oncogene c-Myc as well as with the increased expression of cell cycle inhibitor p21 [319,368,370]. Akt and ERK were other proliferative and survival signaling pathways associated with the actions of biochanin A in PCa cells [368].

In addition, this isoflavone was able to increase the apoptotic rate of LNCaP and PC3 cells [319,368,370,371], which was suggested to be mediated by the TRAIL cell death receptor [371]. Also, biochanin A modulated the expression levels of mitochondrial regulators Bax and Bcl-2, controlling apoptosis (Figure 4) [368].

The administration of biochanin A in vivo reduced the incidence and tumor size in an LNCaP xenograft model [370]. Studies exploring the potential of biochanin A as a possible therapy in PCa are inexistent. However, a report showed that it reduced the PSA production by PC3 cells [372].

#### 6.4.4. Daidzein

Soybean is the major source of daidzein, a natural isoflavone chemically known as 7-hydroxy-3-(4-hydroxyphenyl)-4Hchromen-4-one [373]. Other legumes and fruits can also be important sources of daidzein [373].

Similar to other flavonoids, daidzein has antiproliferative effects in PCa cells (Figure 4). Exposure of PC3 cells to a soy isoflavone concentrate highly enriched in daidzein caused the accumulation of cells in the G2/M phase of the cell cycle, underpinned by the increased expression of p21 [374].

BPH-1, LNCaP, and PC3 cells treated with daidzein exhibited higher apoptotic rates than the control untreated groups [375,376,377]. Concerning the activated apoptotic pathway, this isoflavone increased the expression of DR4 and DR5 death receptors and thus triggered TRAIL-mediated apoptosis [377]. In PC3 cells, treatment with daidzein increased the expression of the proapoptotic Bax protein [375], also implicating the intrinsic apoptosis pathway.

Additionally, several studies have reported that daidzein can reduce PCa growth [378,379,380,381,382] by suppressing migration, invasion, and angiogenesis (Figure 4) [383,384,385].

This isoflavone has been shown to interact with ERβ with some selectivity [364,386], and novel daidzein analogs showed anticarcinogenic activity in PCa cells through ERβ mediation [387]. Also, GPER seems to be involved in the daidzein mechanism of action, with reports existing in other cancer types [388,389]. Further investigation is needed to clarify whether ERs mediate the daidzein effects in PCa.

The remarkable beneficial effects of daidzein counteracting the cancer hallmarks were tested in clinical trials. Men diagnosed with PCa consuming a daily phytoestrogen-enriched diet containing daidzein showed the favorable evolution of PSA levels and free/total PSA ratio [390]. Other significant observed effects included tumor growth inhibition and a chemopreventive action in prostate carcinogenesis, with nontoxic effects [391,392,393,394,395,396,397,398,399,400,401,402,403,404].

#### 6.4.5. Equol

Equol, (3S)-isoflavan-4,7′-diol, is a metabolite of the isoflavone daidzein produced by the activity of intestinal bacteria [405,406]. Only about 30 to 50% of the population can convert daidzein to equol, with a much higher prevalence found in Asian countries compared with Western ones [407,408]. Equol has two diastereoisomers, R-equol and S-equol, with the last one being the natural form. Equol has been shown to have a modest binding affinity for ERs, though it has been suggested that it mimics estrogen’s effects via ERβ and GPER [409,410,411]. Equol was reported to have stronger bioactivity than daidzein and genistein, namely regarding antioxidant activity [412].

The antiproliferative activity of equol in PCa (Figure 4) has been demonstrated in LNCaP, CxR, 22Rv1, and PC3 cells. Equol administration resulted in the inhibition of cell proliferation and diminished cell growth [413,414,415]. Moreover, equol increased the number of LNCaP, LAPC-4, 22Rv1, DU145, and PC3 cells arrested in G0 [413,414]. However, the mechanisms by which equol controls cell proliferation and the dependency on ERs still need clarification.

Equol was also shown to increase the apoptosis of PCa cells (Figure 4) [377,413,414,416]. Despite the information provided by several studies, the mechanisms underlying the proapoptotic effect of equol is highly unknown. However, in LNCaP cells, the presence of this compound was shown to increase the expression of death receptors, specifically the TRAIL receptor [377]. It is also possible to speculate that the mitochondrial pathway can trigger death-induction by equol, as high concentrations of this compound have been related to a significant increment in DNA damage [416].

Although the information available is very limited, equol has been shown to inhibit the invasion of DU145 cells probably by downregulating the expression of MMP-2 and MMP-9 [417].

Reports in animal models and PCa patients also support the beneficial effects of equol delaying prostate tumor growth [391,397,418,419,420,421,422]. Feeding rats with soy flour (containing daidzein, which is converted to intestinal metabolite equol) inhibited the growth of Dunning R3327 prostate adenocarcinoma [391]. In the TRAMP mice, prostate carcinogenesis induced by a high-fat diet occurred concomitantly with the lowered equol serum concentrations because of the adverse effects over equol-producing bacterium [421], which demonstrates the relevance of this bioactive compound in prostate physiology. Equol’s effects include the reduction in PSA levels and blockage of the PSA increase in response to DHT stimulation both in vitro and in vivo [420]. These findings were mirrored in human patients. Men with higher levels of equol in their diet presented reduced PSA levels [397,423] and lower PCa risk and incidence [397,418,422].

#### 6.4.6. Formononetin

The isoflavone formononetin, 7-hydroxy-4′-methoxyisoflavone, can mainly be found as a component of red clover plants [424]. This type of plant is a component of traditional Chinese herbal medicine and has been widely used in China for thousands of years. Formononetin seems to promote cell cycle arrest in PC3 cells by downregulating the Akt pathway and cyclin D1 and CDK4 expression [425]. Moreover, the exposure of LNCaP, DU145, and PC3 cells to formononetin resulted in the induction of apoptotic cell death, which was shown to occur by several mechanisms, namely the augmentation of the Bax/Bcl-2 ratio, suppression of the p38/Akt pathway through the downregulation of the IGF-1/IGF-1R signaling pathway, and the inactivation of ERK1/2 mitogen-activated protein kinase (Figure 4) [426,427,428,429].

A recent study showed a synergistic antitumor effect in PCa cells through the combination of formononetin with docetaxel in one nano-sized drug delivery system [430].

Formononetin can interact with ERβ [364] and GPER [431], with ER-mediated effects reported in breast cancer [432]. No evidence of this relationship exists in PCa, and further research is needed to implicate ERs in the antiproliferative and proapoptotic actions of formononetin over prostate cells.

#### 6.4.7. Genistein

Genistein, chemically known as 4′,5,7-trihydroxyisoflavone, is the main isoflavone present in the human diet, as it is predominantly included in the composition of soybeans, peas, lentils, and other beans [433]. This compound has been shown to interact with both ER isoforms, being one of the most potent phytoestrogens stimulating the transcriptional activity of both ERα and ERβ [364]. However, genistein interaction with ERα was shown to be one-thousandth of the potency of E_2_, whereas for ERβ, the potency was one-third of that of E_2_ [364]. Therefore, it is the selective binding to ERβ that sustains the genistein anticancer activity [364,434,435]. Nevertheless, genistein action through GPER has been reported in other types of cancer except PCa [436,437].

Multiple biological processes in PCa cells have been shown to be modulated by the activity of genistein (Figure 4). This compound seems to decrease the viability and proliferation of LNCaP, 22RV1, DU145, and PC3 cells [380,438,439,440,441,442,443,444,445,446,447,448,449,450], which has been linked to the downregulated expression of cyclin B and the upregulation of the cell cycle inhibitor p21 [438,439,445]. Furthermore, in LNCaP, DU145, and PC3 cells, genistein arrested the cell cycle in G2/M [438,439,440,441,444,445]. Curiously, cell growth inhibition by genistein was associated with suppressing telomerase activity and inhibiting stemness by targeting the hedgehog pathway [442,443]. Another mechanism linked to the genistein anticancer activity was its ability to downregulate oncogene *MDM2* expression, both at the transcriptional and posttranslational levels [451].

Genistein also strongly modulates the survival of PCa cells. It inhibits the activation of the nuclear factor kappa-light-chain-enhancer of activated B cells (NFkβ), mediated by Akt signaling [452]. Genistein also inhibited the radiation-induced activation of the NFkβ pathway in PCa cells [438] and Akt phosphorylation, which resulted in increased apoptosis [444,453]. Another study reported that this flavonoid could abrogate epidermal growth factor (EGF)-induced activation of Akt and inhibit the Akt kinase activity [452].

The reported in vitro findings were mirrored in human PCa cases. By analyzing radical prostatectomy specimens, it was shown that the consumption of genistein was related to the increase in apoptotic rate [394,453], together with the upregulation of p53 [453].

Genistein can also control the metastatic spread of PCa cells. This natural compound has been demonstrated to inhibit the contact-stimulated migration of PCa cells [383,444,454] and suppress MMP-2 activity and cell invasion [444,455,456,457,458]. Furthermore, it reversed the EMT in PCa cells by increasing E-cadherin expression and reducing that of vimentin at the mRNA and protein level [459,460]. Moreover, genistein-treated DU145 and PC3 cells displayed a decreased activity of focal adhesion kinase [461], which was suggested to control the aggressiveness of PCa [462].

Additionally, the angiogenic process in PCa seems to be modulated by genistein, since treatment with this flavonoid suppressed VEGF expression [463,464].

Interestingly, the stimulation of LNCaP, LAPC-4, VCap, and PC3 cells with genistein also decreased or even inhibited PSA expression [465,466]. 

Genistein, like other plant-derived compounds, can induce epigenetic alterations in human cells, altering the landscape of active molecular targets [467]. In PCa, studies showed that the exposure of LNCaP and LAPC-4 cells to this natural agent reduced the methylation of the ERβ promoter, thus increasing the receptor expression [468], which can be an additional mechanism enhancing the estrogenic effects of genistein.

This phytoestrogen can also modulate DNA methylation in the promoter regions of retinoic acid receptor β (*RARβ2*), glutathione S-transferase P1 (*GSTP1*), Ras association domain family 1 (*RASSF1A*), and ephrin B2 (*EPHB2*) genes, which seems to be protective against PCa [469,470]. Moreover, this natural compound increased the expression of the tumor suppressor gene *BTG3* in the RWPE-1, DU145, and PC3 cells as well as the *sFRP1* and *Smad4* genes via demethylation and histone modification [444,471].

Noteworthy, genistein has also been reported to modulate the expression of regulatory microRNAs. It reduced the expression of the oncogenic miRNAs miR-1260b and miR-151, which are upregulated in PCa [444,472]. On the other hand, genistein treatment increased the tumor suppressor miR-574-3p, which is downregulated in PCa [473].

Studies in animal models have confirmed the beneficial properties of dietary genistein in suppressing the development of PCa [474,475,476,477]. A low-fat diet with soy protein and isoflavones, namely genistein, reduced the tumor growth in LNCaP xenografts [478]. Furthermore, genistein in the diet seems to reduce the incidence of poorly differentiated PCa and improve survival in TRAMP mice [479,480,481,482]. This antitumor role of genistein is supported by reports that showed a reduction in the proliferation and increase in apoptosis in the ventral prostate of Simian Virus-40 T-antigen (SV-40 Tag) targeted probasin promoter rat model, a transgenic model developing PCa spontaneously [476], and in the cancerous prostate dorsolateral prostate of Lobund-Wistar rats [475]. At the molecular level, genistein reduced the cyclin D1 levels post-transcriptionally, disrupting cell cycle progression, and thus reducing proliferation [479]. One of the molecular mechanisms related to these genistein effects is PTEN/Akt axis regulation. Genistein in the diet upregulated PTEN, inhibiting the activation of Akt and restoring the activation of GSK-3β [475,479]. Furthermore, genistein in the diet retained cadherin-1 expression through the decrease in snail-1 transcription [479], which can be critical in maintaining the integrity of prostatic epithelial cells and retarding cancer progression.

Curiously, this natural compound reduced the AR mRNA and protein levels [475,483] as well as ERα and ERβ expression in the rat prostate [483]. Moreover, genistein was shown to downregulate the AR levels in LNCaP cells through the activity of ERβ [484], which highlights that aside from their direct actions, estrogens could modulate the response to androgens. In addition, genistein decreased steroid receptor coactivator-3 (SRC-3) levels in the ventral prostate of SV-40 Tag rats [476]. SRC-3 is an important regulator of PCa proliferation and survival and is essential for the progression of prostate tumorigenesis in the TRAMP model [485]. Another interesting effect of genistein is its ability to reduce intraprostatic DHT levels together with the diminished expression of 5α-redutase-2 [474], corroborating the benefit of genistein to prevent prostate diseases such as PCa.

The protective role of genistein against PCa development is also supported by the reduction in IGF-1 [474,476], a player that has been associated with the increased risk of an advanced stage of PCa [486], and the inhibition of osteopontin [480], an extracellular matrix protein secreted by infiltrating macrophages and prostate tumors cells themselves [487,488,489], which is related to increased tumorigenicity and metastatic ability [490].

From a therapeutic perspective, the beneficial effects of genistein, when joined with classical chemotherapy and antiandrogenic drugs, are worth noting. In fact, this isoflavonoid has been tested in clinical trials with PCa patients with promising results [397,491,492,493,494,495,496,497].

The combination of genistein with polysaccharides enhanced the activity of docetaxel, bicalutamide, and Src kinase inhibitors in both androgen-sensitive and CRPC cells, namely, in LNCaP, CWR22Rv1, and PC3 cells [498]. Furthermore, this natural agent increased the efficacy of cabazitaxel in C4-2, ARCaP_M_, and PC3 cells [499] and paclitaxel in LNCaP and DU145 cells [500]. Genistein has also been shown to potentiate the effect of radiation, diminishing PCa cell proliferation and tumor growth [501,502,503,504,505]. The use of BIO 300, a nanosuspension of genistein, sensitized human PCa xenografts to radiation therapy [506].

Additionally, several reports have demonstrated that genistein combined with SB715992, an experimental inhibitor of kinesin spindle proteins that play an essential role in mitotic spindle formation, can improve the outcomes in PCa patients [507].

A limitation found in clinical trials for using genistein in treatment is its reduced bioavailability upon oral delivery. Using nanostructured delivery systems carrying genistein is a possible solution to increase its bioavailability and enhance anticancer action against PCa [508]. Some approaches have been tested with good results, namely, a nanoliposomal formulation encapsulating celecoxib and genistein was shown to inhibit the cyclooxygenase-2 (COX-2) pathway and GLUT1 expression, preventing PCa cell proliferation [449]; another liposomal formulation containing plumbagin and genistein inhibited proliferation and induced the apoptosis of PCa cells [509,510], while nanoparticles loaded with genistein and doxorubicin reduced the development of PCa metastasis by amplifying oxidative stress damage [511], and genistein-gold nanoparticle conjugates displayed antioxidant and antitumorigenic effects in PCa [512].

Despite genistein’s beneficial effects against PCa, reports that demonstrated a dual effect of this compound in PCa cells and in vivo [513,514,515,516,517] should be given attention. Moreover, it is critical to deeply understand the mechanisms underlying genistein actions in PCa and establish the ideal threshold dose to avoid dual effects before disseminating its use in treatment.

#### 6.4.8. Naringenin

Naringenin, 4′,5,7-trihydroxyflavanone, is a bioactive compound mainly found in grapefruit and orange [518,519], which has been demonstrated to have antiproliferative and proapoptotic activity in PCa cells (Figure 4) [520]. Information concerning the effects of naringenin and its mechanism of action in PCa is still scarce. However, naringenin-reduced oxidative stress [521] and induced apoptotic cell death mediated by the PI3K/Akt and MAPK signaling pathways [520]. This biologically active substance is also able to repress the migration and invasion of PCa cells by increasing the expression of E-cadherin and diminishing the vimentin expression levels [522,523]. On the other hand, the exposure of LNCaP cells to naringenin stimulated DNA repair, preventing mutagenic changes as a consequence of oxidative damage [524], which raises curiosity about the role of this compound against early events in human prostate carcinogenesis.

Naringenin interaction with ERs has been demonstrated, with a stronger binding affinity and higher transcriptional activity for ERβ [364]. It has also been shown to modulate the receptors’ expression levels in breast tumors, decreasing ERα expression while increasing ERβ [525]. Thus, it is reasonable to assume that the actions of naringenin in PCa could be mediated by ERs, with a relevant role for ERβ. Additional investigation is needed to clarify this relationship and further explore the actions of naringenin.

#### 6.4.9. Kaempferol

Kaempferol, 3,4,5,7-tetrahydroxyflavone, is a secondary metabolite found in many plants, plant-derived foods, and traditional medicines [526], which seems to have an important role against PCa (Figure 4). The beneficial effects of kaempferol have been demonstrated by a set of in vitro and in vivo experimental evidence [527,528,529,530,531]. Several studies have reported the antiproliferative effects of kaempferol [381,532,533] by increasing the proportion of PCa cells blocked in the G2/M phase [381,533] and its ability to inhibit the DHT-stimulated growth of LNCaP cells [532,534,535]. Moreover, the proapoptotic properties of kaempferol have been described [530,532,536,537]. This compound augmented the apoptosis of PCa cells, which was accompanied by the increased expression of TRAIL, enhanced levels of caspase-8, caspase-9, and caspase-3, and the inhibition of the NFkβ pathway [530,536,537].

A study addressing the effect of a mix of flavonoid compounds including kaempferol in a preclinical murine model demonstrated the ability of these compounds to target AR signaling and inhibit PCa growth [531]. Despite the known selectivity of kaempferol for ERs [364], no ER-mediated effects were reported in PCa. Nevertheless, as shown in other tissues, it seems to mainly act by antagonizing ERα activity [538,539].

#### 6.4.10. Myricetin

Myricetin, 3,3′,4′,5,5′,7-hexahydroxyflavone, is a family member of flavonoids normally found in vegetables, fruits, nuts, berries, tea, and red wine [540,541]. This phytoestrogen has recognized antioxidant properties [542], and has been demonstrated to inhibit proliferation and induce apoptosis in PC3 cells [543]. Moreover, myricetin effectively suppressed the in vivo progression of PCa by disrupting the interaction of the protooncogene protein proviral integration site for Moloney murine leukemia virus-1 (PIM1) with C-X-C chemokine receptor type 4 (CXCR4) [544]. PIM1 is overexpressed in PCa, playing an important role in tumorigenesis, castration resistance, and metastasis [545,546,547,548,549]. PIM1 promotes the phosphorylation and surface expression of CXCR4, supporting the CXCL12–CXCR4 axis responsible for cancer cell proliferation and metastasis [547,550].

An interesting study showed that PLGA-encapsulated myricetin formulation combined with enzalutamide enhanced the effects of enzalutamide alone in a xenograft model of PCa [551].

This compound was shown to bind ERs competing with E_2_ and was able to reduce ERα expression in breast cancer cells [552,553]. Nevertheless, there is no evidence of the involvement of ERs in mediating the myricetin effects in PCa.

#### 6.4.11. Quercetin

Quercetin, also known as 3,3′,4′,5,7-pentahydroxyflavone, is a polyphenol belonging to flavonoids and is commonly found in several fruits and vegetables, namely capers, lovage, dill, cilantro, onions, apples, and berries [554]. The main biological properties of this phytoestrogen include antioxidant, antiallergic, anti-inflammatory, and antiviral activities [555,556,557]. Additionally, it has been proven that quercetin can be useful against several types of cancer such as lung, liver, breast, colon, cervical, and prostate [309,558].

Quercetin can reduce the viability and proliferation of LNCaP, LAPC-4, DU145, and PC3 cells [533,559,560,561,562,563,564] in part by diminishing the action of growth factors [565,566]. Quercetin has been shown to arrest cell cycle progression in all phases of the cycle [559,564,567,568]. In LNCaP cells, quercetin arrested the cell cycle in the S phase, significantly reducing the number of cells in G1 [564]. The control of cell cycle progression and cell proliferation was shown to depend on the regulation of several molecular targets (Figure 4). Quercetin strongly inhibited the expression of cyclin B1 and CDK1 and increased the levels of p21 and hypophosphorylated Rb protein in a dose-dependent manner in PC3 cells [559,568] and targeted the PI3K/Akt signaling pathway [569]. Moreover, the inhibition of PCa growth by quercetin can be mediated by the reduction in AR signaling, as quercetin demonstrated the ability to suppress AR expression and inhibit receptor function [561,562,566,570]. Considering ERs, although quercetin has been shown to act through these receptors in other tissues [364,436,571], no reports exist concerning the involvement of ERs in PCa cell responses.

Research efforts have been made to clarify quercetin’s actions and the molecular players involved in stimulating PCa cell apoptosis. Quercetin induced the TRAIL-mediated apoptosis of LNCaP, DU145 and PC3 cells by increasing the expression of TRAIL and DR5 cell death receptors [536,572,573]. Moreover, the exposure of PCa cells to quercetin promoted the activation of caspase-3, caspase-8, and caspase-9 and affected the expression of several apoptosis-related proteins. It decreased the levels of heat shock protein 90 [574] and antiapoptotic proteins Bid and Bcl-2 and increased the expression of the proapoptotic protein Bax [543,567,572,575,576,577,578].

Another mechanism reported to diminish the survival of DU145 cells in response to quercetin is the induction of ROS production, which will lead to an increase in oxidative stress, culminating with cell death [579].

Quercetin also affects PCa cell migration and invasion, stemness, and angiogenesis [580,581]. It synergizes with epigallocatechin gallate to inhibit PCa stem cell characteristics, EMT, invasion, and migration [582]. Accordingly, the expression of prostate stem cell antigen is downregulated by quercetin in DU145 cells [583,584]. Moreover, quercetin decreased the migration of PC3 cells induced by TGF-β [585]. In PC3 cells, quercetin also reversed the EGF-induced EMT and invasiveness via activation of the EGFR/PI3K/Akt pathway [586] and downregulated the expression of MMP-2 and MMP-9 [587]. Furthermore, this phytoestrogen demonstrated the capability of reducing angiogenesis by targeting the VEGFR-2-regulated Akt/mTOR signaling pathways [588].

Interestingly, the actions of quercetin also seem to target the immune response, which can be highly relevant for treatment purposes, particularly considering immunotherapy. It stimulated GM-CSF production in PC3 cells, and the conditioned medium of quercetin-treated PC3 cells increased the chemotaxis of human dendritic cells, suggesting that quercetin treatment can promote the recruitment of dendritic cells to the tumor site [589].

Noteworthy, quercetin sensitized PCa cells to chemotherapy and antiandrogenic drugs. In combination with docetaxel, paclitaxel, doxorubicin, or enzalutamide, quercetin increased the sensitivity of PCa cells to treatment, leading to a notorious anticarcinogenic effect compared with the administration of the anticancer drugs alone both in vitro and in vivo [44,45,46,47,590,591,592].

Several robust findings have pointed out the mechanisms that stimulated the effects of chemotherapeutics in suppressing cell proliferation and invasion, increasing apoptosis, and reducing tumor growth in the presence of quercetin such as the inhibition of the PI3K/Akt and signal transducer and activator of transcription (Stat) 3 signaling pathways, increased expression of cleaved caspase-7 [46], activation of the mitochondrial/ROS pathway, decreased expression of the multidrug resistance-related protein (MRP1) [45], and reduced blood concentrations of growth factors like VEGF and EGF [46].

Resistance to the next-generation antiandrogen, enzalutamide, has been proposed to depend to a large extent of the *AR* splice variant AR-V7 [593]. This constitutively active variant is generated by the alternative splicing of the AR involving the activity of splicing factors such as hnRNPA1 [594,595]. Quercetin reduced the expression of hnRNPA1, and consequently, that of AR-V7, sensitizing resistant PCa cells to enzalutamide treatment [47]. Furthermore, quercetin can reverse docetaxel-resistance in vitro and in vivo through the AR and PI3K/Akt signaling pathways, supporting its clinical use in docetaxel-resistant PCa [596]. Quercetin also increased PCa radiosensitivity by targeting the radiation-induced ARv7 [597], supporting the possibility of applying radiotherapy combined with quercetin in PCa treatment. Studies in rodents further confirmed the potential of quercetin to prevent PCa growth by suppressing cell survival and proliferation and the expression of antiapoptotic proteins [598,599]. Quercetin supplementation reverted the augmented levels of proliferative and antiapoptotic markers as well as the decreased expression of proapoptotic markers resultant of N-methyl-N-nitrosourea plus testosterone induction of prostate carcinogenesis [598]. In this carcinogen-induced PCa model, quercetin also increased the expression of antioxidant enzymes and decreased the expression of insulin-like-growth factor receptor 1(IGFIR), Akt, and AR [598].

The beneficial use of quercetin was also reported in PCa patients [39,40]. One study evaluated the association between serum vitamin D and dietary quercetin and PCa risk in African men. In individuals with vitamin D deficiency, the augmented levels of quercetin in diet were linked to a lower risk of PCa [40]. It has also been reported that metastatic PCa patients receiving increasing doses of quercetin displayed longer PSA doubling time [39]. However, the enormous benefits of quercetin in suppressing PCa growth have some limitations in clinical practice and therapy. As with other natural compounds, quercetin displays reduced water solubility and low bioavailability, which largely conditions the potential of therapeutic application. To solve this problem, a few studies have evaluated the use of nanoparticles to deliver quercetin directly to PCa cells. The use of cationic PEGylated niosome-containing quercetin and quercetin-loaded nanomicelles assembled from DSPE-PEG_2000_ showed promising results regarding tumor regression [600,601]. More recently, several approaches such as LHRH-conjugated, PEGylated, poly-lactide-co-glycolide nanocapsules [602], carboxylated graphene oxide as a nanocarrier [603], chitosan-coated, quercetin-loaded PLGA nanoparticles [604], and others [605] have shown promising results in controlling PCa growth and progression, opening up new avenues of research for exploiting treatment approaches.

#### 6.4.12. Coumestrol

Coumestrol, 3,9-dihydroxypterocarp-6a(11a)-en-6-one, is a natural organic compound found in soybean, legumes, Brussels sprouts, alfalfa, and spinach [606,607]. Recently, this natural compound was shown to exhibit anticancer activity against several types of cancer [608,609]. However, only a few studies have investigated the effects of coumestrol in PCa cells. Treatment with coumestrol inhibited the proliferation of LNCaP and PC3 cells and stimulated apoptosis by inducing DNA damage and mitochondrial dysfunction as a consequence of the loss of mitochondrial membrane potential and increased ROS production [416,610]. Furthermore, PCa cells treated with coumestrol showed elevated levels of the proapoptotic protein Bad and cleaved caspase-3 and caspase-9 [610].

The antiproliferative and prosurvival properties of coumestrol seem to be mediated by the modulation of the PI3K/AKT and ERK1/2 and JNK MAPK cell signaling pathways, which have also been implicated in the decreased migration of LNCaP and PC3 cells in the presence of this phytoestrogen (Figure 4) [610]. Furthermore, the capability of coumestrol in reducing PCa progression can be related to HIFα suppression. In hypoxic PC3 cells, coumestrol suppressed HIFα by inhibiting ROS-mediated sphingosine kinase 1 [611], a key enzyme that converts sphingosine into sphingosine 1-phosphate [612], a sphingolipid metabolite and a lipid mediator crucial in several biologic processes of carcinogenesis [613,614,615].

In humans, coumestrol intake is associated with a reduced risk of developing PCa [616]. Of note, coumestrol’s actions have been related to its ability to bind ERs, with the capability of the transcriptional activation of both ERα and ERβ at concentrations of 1–10 nM [364,617]. Indeed, like genistein, coumestrol is among the phytoestrogens with a higher estrogenic potency in transactivation assays compared with E_2_ [364]. In PCa, evidence for the ER-mediated effects of coumestrol is scarce. Nevertheless, the intake of phytoestrogens like coumestrol substantially reduced the PCa risk among men with specific polymorphic variation in the promoter region of the *ERβ* gene [618], which is suggestive of this receptor’s involvement.

#### 6.4.13. Resveratrol

Resveratrol, also known as trans-3,40,5-trihydroxystilbene, is a stilbene polyphenol highly abundant in red grapes, peanuts, and other fruits [619]. Generally, red wine still comprises resveratrol in amounts ranging from 0.1 to 14.3 mg/L [620].

This natural agent is considered as a phytoestrogen due to its ability to bind ERs and GPER, competing with natural estrogens and altering the normal biological response including an anticarcinogenic effect [621,622,623]. Several studies have shown that resveratrol targets cancer cells without producing harmful effects on nonmalignant cells [48,49,624,625,626]. For this reason, it has been considered as an ideal molecule for anticancer therapy.

Resveratrol has obtained attention as a chemopreventive agent targeting multiple signaling pathways and affecting several cellular processes, namely cell viability, proliferation, apoptosis, survival, migration and invasion, and metabolism. A large number of studies have shown that resveratrol reduced the viability and proliferation of both androgen-sensitive and CRPC cells, namely, LNCaP, 22Rv1, DU145, and PC3 cells. [256,627,628,629,630,631,632]. It increased the number of cells in the G0/G1 arresting phase and diminished the number of cells in the G2 phase [630,631,632], likely by reducing the expression of cyclin D1, B1, and E and increasing the expression of some cell cycle inhibitors such as p21 and p27 [631,633]. Interestingly, resveratrol seems to reduce PC3 and 22Rv1 cell survival without affecting nonmalignant PNT1A cells [631]. In fact, the antiproliferative effect of resveratrol has been associated with its ability to reduce ERα and increase ERβ expression [634,635]. Other mechanisms associated with the effects of resveratrol in reducing PCa cell proliferation include the inhibition of the: (i) AR and CXCR4 pathway [625]; (ii) nuclear factor NFkβ, which is also associated with cell survival; and mTORC1 pathway, which promotes cancer cell growth, survival, invasion, and angiogenesis [636,637].

The induction of PCa cell apoptosis by resveratrol is dependent on modulating the expression of the pro- and antiapoptotic proteins (Figure 4). Resveratrol can upregulate the expression of proapoptotic genes *BAX*, *BID*, and *BAK* and downregulate the antiapoptotic ones, *MCL-1*, *BCL-2*, and *BCL-XL*, in several human and rodent PCa cell lines, namely, LNCaP, C4-2B, DU145, PC3, as well as TRAMP-C1, TRAMP-C2, and TRAMP-C3 [633,638,639,640,641]. The consequent alterations in the apoptosis-related proteins culminated with the increased activity of several caspase family members, namely, caspase-7 and caspase-3 [256,632,638,639,641,642]. Furthermore, resveratrol sensitized the LNCaP cells and PC3 xenografts to TRAIL-mediated apoptosis by increasing the expression of TRAIL receptors [643,644]. Other mechanisms involved in resveratrol-induced apoptosis include the increased expression of p53, the generation of ROS, the downregulation of the PI3K/Akt survival pathway, and autophagy induction [633,638,639,640,645]. Moreover, the activation of apoptotic targets was also achieved by the activation of FOXO transcription factors [643].

Concerning PCa progression, resveratrol was shown to control cell migration, invasion, and EMT. Resveratrol treatment reduced the migration and invasion of LNCaP, DU145, PC3, PC3M-MM2, and MAT-LyLu cells [627,646,647,648]. Moreover, this natural agent could revert EMT in LNCaP and PC3 cells by increasing the expression of the epithelial marker E-cadherin, and decreasing the expression of vimentin, a mesenchymal marker [649]. In vivo studies in TRAMP and SV-40 Tag rats confirmed the results obtained in cell lines and the valuable effects of resveratrol in suppressing PCa growth [476,635].

The effect of resveratrol extends to other features of cancer cells such as metabolic rewiring and the capacity to survive in hypoxic microenvironments. Resveratrol has been shown to inhibit *HIFα* expression, repressing the progression of LNCaP xenograft tumors [650]. Furthermore, in hypoxic LNCaP cells at low androgen levels (mimicking CRPC), resveratrol inhibited hypoxia-induced nuclear accumulation of β-catenin, inhibiting its mediated AR signaling [650]. Concerning the metabolic response, resveratrol decreased the uptake of glutamine and deregulated lipid metabolism in human (LNCaP, DU145, PC3, C4-2B) and murine (TRAMP and HMVp2) PCa cells as well as in LNCaP and HMVP2 xenografts [639,650,651], which delayed the progression of PCa.

Overall, due to all the above-mentioned beneficial properties of resveratrol, several approaches have been developed to test its therapeutic effectiveness, alone or in combined therapy, together with the optimal delivery method, using different coated nanoparticles targeting PCa cells [652,653,654,655,656,657]. Alginate nanoparticles containing resveratrol and curcumin exhibited cytotoxic effects on DU145 cells [652]. Also, resveratrol-loaded poly(lactic-co-glycolic acid) nanoparticles reduced the LNCaP cell viability, arrested cell cycle at the G1-S transition phase, enhanced ROS levels, and promoted apoptosis, causing the externalization of phosphatidylserine, DNA nicking, and the loss of mitochondrial membrane potential [657]. Other similar resveratrol-loaded nanoparticles based on a poly(epsilon-caprolactone) and poly(d,l-lactic-co-glycolic acid)–poly(ethylene glycol) blend also displayed cytotoxicity in LNCaP, DU145, and PC3 cells [653], and resveratrol-loaded poly(2-hydroxyethyl methacrylate)-chitosan-based nanoparticles also showed cytotoxicity in PC3 cells [658]. Planetary-ball-milled nanoparticles encapsulated with resveratrol in combination with docetaxel and conjugated with folic acid increased the apoptotic cell number in PC3 and docetaxel-resistant PC3 cells, reducing the expression of *NF-kB p65*, *COX-2*, and pro- (*BAX*, *BAK*) and antiapoptotic (*BCL-2*, *BCL-XL*) genes and downregulating survivin as well as increasing cleaved caspase-3 expression [654]. Moreover, functionalized mesoporous silica nanoparticles encapsulating resveratrol displayed antiproliferative effects and sensitized PCa cells to docetaxel in a hypoxic cell environment [655].

The combination of resveratrol with docetaxel was also tested using liposomes and showed antitumor efficiency against PCa, reducing proliferation and increasing apoptosis [659]. Resveratrol-loaded solid lipid nanoparticles were another approach tested in PCa cells as a potential carrier for delivery chemotherapeutics at the tumor site [660]. Other combinations, even without using nanocarriers, showed the enhanced anticarcinogenic effects of cisplatin, docetaxel, doxorubicin, and resveratrol in PCa cells [661,662,663].

Various reports have also shown that resveratrol enhances the response to ionizing radiation in LAPC4, DU145, and PC3 cells [631,664,665,666,667,668].

The results obtained with resveratrol in preclinical studies support testing this phytoestrogen in clinical trials concerning its safety, tolerability, and effective dose in PCa patients [39,41]. A pilot study assessing the effects of a phytotherapeutic intervention containing turmeric, resveratrol, green tea, and broccoli sprouts revealed that its use is feasible in men with biochemically recurrent PCa and a moderate PSA rise rate [41]. In a phase I study in nonmetastatic biochemically recurrent PCa patients, 4000 mg of a pulverized muscadine grape skin containing ellagic acid, quercetin, and resveratrol was safe in augmenting PSA doubling time, with no serious adverse effects being reported in the study time-frame [39]. This formulation was further investigated in a randomized, multicenter, placebo-controlled, dose-evaluating phase II trial. However, no significant difference in PSA doubling time was observed between the control and treatment groups [669].

Besides the beneficial effects of resveratrol and the promising results from preclinical studies and clinical trials, it remains crucial to better understand its detailed mechanism of action and the ideal dose as a therapeutic agent against PCa.

#### 6.4.14. Diosgenin

Diosgenin, also known as 25R-spirost-5-en-3β-ol, is a steroidal sapogenin with a structure similar to cholesterol and other steroids, being used by the pharmaceutical industry in the synthesis of sex hormones and corticosteroids for application as anti-inflammatory, androgenic, and contraceptive drugs [670,671]. This natural compound is commonly found in a panoply of plants, namely, in species from the *Dioscorea*, *Trigonella*, *Costus*, and *Smilax* genera, particularly in tubers and seeds [672,673,674,675].

This steroidal sapogenin has been shown to have antimicrobial, antioxidant, anti-inflammatory, antidiabetic, and antiobesity activity [676]. Furthermore, diosgenin’s role in cancer is well-described. It has a potential antitumor effect in many types of cancer, namely, in leukemia [677,678,679,680] and gastric [681,682], lung [683,684,685], breast [686,687], liver [688,689,690], renal [691], colon [692,693,694], pancreas [695], bone [696], cervical [697], skin [698,699], and oral [700] cancers.

The anticancer effects of diosgenin have also been confirmed in PCa (Figure 4) [701,702,703]. This sapogenin reduced the PC3 cell viability in a dose-dependent manner [703]. Along the same line, diosgenin and a fenugreek extract containing diosgenin as the bioactive compound inhibited the proliferation of PCa cells [702,704], induced cell cycle arrest in the G2/M phase, and increased the sub-G1 and G2/M phase cell population [704,705].

Diosgenin also augmented apoptosis in DU145 and PC3 cells [702,704], underpinned by the increased expression of caspase-9 [702]. The fenugreek extract also reduced the expression of the mutant p53 protein in DU145 cells [705]. In addition, diosgenin can activate autophagy [702], a cell death process distinct from apoptosis that contributes to the maintenance of the stability of the intracellular environment and the normal physiological functions of the cell, which have been related to tumor growth inhibition [706,707,708]. DU145 cells treated with diosgenin showed a large number of autophagosomes including autophagosomes containing mitochondria [702].

The molecular mediators of diosgenin’s actions in suppressing cell survival and growth have been explored. In PC3 cells, diosgenin increased the expression of cell cycle inhibitor p21 and inhibited the phosphorylation of EGFR [705]. Furthermore, it was confirmed that diosgenin inhibits several signaling pathways, namely, the PI3K/Akt, JNK, and ERK pathways [701,702,709]. Exposure of PCa cells to diosgenin was shown to reduce PI3K, Akt, mTOR, JNK1/2 and ERK1/2 phosphorylation [701,702,705,709] by regulating the TGF-β and the HGF pathways.

Concerning tumor progression, the steroid sapogenin diosgenin reduced the migration and invasion capabilities of PC3 cells [701,704], inhibiting the expression and activation of MMP-2, MMP-7, and MMP-9, which have a crucial role in extracellular matrix degradation [701]. In DU145 cells, diosgenin inhibited HGF-induced motility, invasion, and EMT by increasing E-cadherin and decreasing vimentin and Mdm2 expression [709]. The role of diosgenin in inhibiting PCa tumor progression is also supported by its ability to suppress angiogenesis, as it blocked the expression of VEGF in PC3 cells and the tube formation of endothelial cells [701].

Few studies have investigated the anticancer activity of diosgenin in animal models. However, a study showed that diosgenin treatment diminished the prostate-to-body weight ratio and serum PSA levels in a rat model of BPH, established by the subcutaneous injection of testosterone propionate [710]. Reduced prostate size could be related to a proapoptotic effect of diosgenin, increasing Bax and p53 expression, and reducing the expression of Bcl-2 [710]. Furthermore, diosgenin-treated rats displayed an elevated activity of SOD and GPx and reduced the MDA levels [710], suggesting that diosgenin can mitigate oxidative stress in the hyperplastic prostate. Moreover, diosgenin reduced the tumor growth and metastasis in xenograft [711] and TRAMP mice by regulating NF-κB/STAT3 signaling [712].

Regarding the panoply of diosgenin effects against PCa, modulating many cellular processes, several studies evaluating the effect of diosgenin analogs and/or derivatives have emerged. Most of these compounds were revealed to have antiproliferative and proapoptotic activity in PCa, namely, in DU145 and PC3 cells [713,714,715,716,717], with some displaying advantages relative to diosgenin.

## 7. Lessons Learned, Conclusions, and Future Directions

Estrogens, the female sex hormones, have been shown to play a role in men’s physiology. The adult prostate is one of the male reproductive organs that is highly dependent on the actions of estrogens, which oscillate from anticarcinogenic, supporting the use of these hormones in therapy, to protumorigenic, accelerating PCa growth. However, the relationship between PCa risk and E_2_ serum levels remains controversial, with most studies not finding statistically significant associations. This lack of relationship can be a consequence of the broad range of serum E_2_ concentrations found in men, which is a result of a set of different variables not always controlled for in the different studies. E_2_ concentrations have been shown to vary with age group, ethnicity, and endocrine and metabolic conditions including adiposity and body mass index as well as with specificities in aromatase expression and activity. The intraprostatic production of estrogens cannot be ignored in the scenario of their influence on prostate physiology and carcinogenesis. External androgen precursors or circulating testosterone can be converted to estrogens locally in the prostate by aromatase. Indeed, it has been shown that increasing the aromatase activity with augmented E_2_ production accompanies the development of PCa (noncancerous prostate < primary tumors < metastatic tumors). Nevertheless, further research is needed to clarify the impact of intraprostatic estrogen biosynthesis in prostate pathology, as clinical trials using aromatase inhibitors have failed to confirm its utility [718,719]. Additionally, it would be reasonable to assess the effect of estrogen therapy in men with prostate cancer and low or high circulating estrogen levels in an analogy to breast cancer therapy in pre- and postmenopausal women.

As mentioned, and after the discontinuity of using estrogens in PCa treatment, research efforts over the years have produced a vast amount of information establishing the dual role of estrogens in prostate carcinogenesis, balancing from guardians to guilty. The enormous amount of data herein reviewed have detailed the biological processes and molecular events orchestrating the actions of estrogens as prostate carcinogens. Naturally occurring and synthetic hormones can augment cell proliferation, resistance to apoptosis, migration and invasion, and other events involved in tumor growth and progression. These procarcinogenic effects have mainly been related to the differential activation of ERα. In contrast, the role of estrogens in counteracting PCa development and metastization by reducing cell growth, promoting cell cycle arrest, increasing apoptosis, and suppressing migration, invasiveness, and angiogenesis has mainly implicated ERβ as the mediator. Nevertheless, the complexity of the actions of estrogens is far from being enlightened. For example, it remains to be disclosed whether differential responses in human patients may rely on the physiological condition, which may disrupt ERα and ERβ expression, changing prostate cells to a cancer-like phenotype. This hypothesis is gaining strength considering the reports showing that ER expression is altered in the prostate of diabetic [720] and obese [721] patients. Of note, diabetes markedly reduced ERβ and GPER expression in PCa patients [720].

More research is also needed to clarify the role of GPER in PCa, as the last decades have witnessed the emergence of reports showing that it can trigger both pro- and anticancer responses. It will also be of paramount importance to further investigate the relationship between GPER, aromatase expression, and intraprostatic estrogen production.

Another relevant issue in the landscape of prostate responses to estrogens is related to the fact that different concentrations/doses of estrogens can trigger distinct and sometimes opposing effects. Generally, data support that lower concentrations/doses are associated with the procarcinogenic effects of these hormones, with the higher doses being antitumorigenic. This was the basis of estrogen therapy in PCa, with the high hormone doses causing well-characterized adverse effects. A great pharmacological challenge in PCa therapy relies on developing new approaches allowing for the use of reduced doses of estrogens, receptor-specific agonists, or alternative estrogen-like compounds with similar properties to estrogens, but fewer side effects. In recent years, many studies have investigated the effect of natural compounds with estrogen-like activity in controlling PCa. This review summarized the promising properties of a panoply of phytoestrogens against PCa, reported both in vitro and in vivo. Overall, distinct substances of this class of compounds have been shown to control PCa cell proliferation and tumor growth, reduce migration and invasion, suppress EMT, angiogenesis, and metastization, and also regulate the bioenergetic metabolism of PCa cells. Several molecular targets have been discovered alongside identifying the actions of phytoestrogens and disclosing their mechanisms of action. These effects encompass the reduction in cyclins and CDK expression, the increased levels of p53 and decrease in those of p21, the activation of TRAIL receptors and caspase cascades, the reduction in β-catenin and MMP expression, and the inhibition of the Akt/PI3K and NFκB pathways, while activating MAPK signaling. Based on their tumor-suppressor properties, phytoestrogens such as chrysin, daidzein, genistein, quercetin, coumestrol, resveratrol, and equol have been pointed out as possible alternative therapies for PCa, and have been tested on humans. Equol, quercetin, coumestrol, and resveratrol are among the most promising agents as they have been shown to reduce the PSA levels and lower PCa risk and incidence when used in the diet. It is also quite challenging to demonstrate that quercetin can promote the recruitment of dendritic cells to the tumor site, which opens up new perspectives of research for evaluating phytoestrogens as anticancer molecules.

In summary, the discussion in the present review systematized the role of estrogens in prostate carcinogenesis and provided the scientific fundamentals for the evolution of PCa treatment based on the use of natural estrogenic substances. Using natural bioactive compounds such as phytoestrogens in PCa therapy is an exciting and powerful research and clinical question. Even if these compounds are not chosen as a first-line therapy, they can be combined with conventional treatments such as chemotherapy, radiation therapy, and immunotherapy, increasing their efficacy and/or mitigating the systemic adverse effects. Ongoing research efforts and others yet to come will expand the treatment options available for PCa patients.

## Supplementary Materials

The following supporting information can be downloaded at: https://www.mdpi.com/article/10.3390/biomedicines12081636/s1, Table S1: Anti-proliferative and pro-apoptotic effects of estrogens in prostate cells; Table S2: Phytoestrogens with a role in PCa.
