# Supplementary material for: The Pros and Cons of Estrogens in Prostate Cancer: An Update with a Focus on Phytoestrogens
Source: Biomedicines. 2024 Jul 23;12(8):1636. doi: 10.3390/biomedicines12081636 (PMC11351860; doi:10.3390/biomedicines12081636)
Supplement: Supplementary file 1 [file biomedicines-12-01636-s001.zip › Supplementary Table S1.pdf]

Supplementary Table S1. Anti-proliferative and pro-apoptotic effects of estrogens in prostate cells.

Type of study	Cell line/ Animal model	Compound	Concentration/Dose	Assay model/ Method of administration	Time of treatment	Assay	Effect	Activated pathway	Ref.
in vitro	PrECs	2-ME$_2$	0.5, 1, 2, 3 and 5 μM	96-well plates<br>6 cm dishes	24 and 48 h	Trypan blue exclusion<br>MTS<br>Flow cytometry	↓ Cell growth<br>↑ Cells in G2/M phase<br>↓ Cells in G1 phase	G2/M arrest	[207]
	BPH-1	8β-VE$_2$*	6 μM	multichambered slides	12 h	Immunohistochemistry	↑ Apoptosis	↑ caspase-3<br>↑ caspase-8	[253]
	RWPE-1	8β-VE$_2$*	6 μM	multichambered slides	12 h	Immunohistochemistry	↑ Apoptosis	↑ caspase-3<br>↑ caspase-8	[253]
	ALVA-31	2-ME$_2$	5 μM	6-well plates<br>6 cm dishes	1 - 6 days	Cell count in haemocytometer<br>Flow cytometry<br>Annexin V-fluorescein isothiocyanate (FITC) and propidium iodide (PI) *in situ*<br>Caspase inhibition	↓ Cell growth<br>↑ Cytotoxicity	-	[208]
	LAPC-4	αE$_2$	0.1 - 10 μM	96-well plates	72 h<br>Up to 20 days	MTS<br>Caspase 3/7 activity	↓ DHT-induced PSA expression	↓ DHT-induced cyclin A and cyclin D1 expression	[200]

						↓ DHT-induced cell growth<br><br>↓ DHT-induced DNA biosynthesis		
LAPC-4	βE2	0.1 - 10 μM	96-well plates	72 h<br>Up to 20 days	MTS<br><br>Caspase 3/7 activity	↓ DHT-induced PSA expression<br><br>↓ DHT-induced cell growth<br><br>↓ DHT-induced DNA biosynthesis	↓ DHT-induced cyclin A and cyclin D1 expression	[200]
LAPC-4	E2	10^{-8} - 1 μM	96-well plates	72 h	MTT<br><br>Caspase-3 activity<br><br>ROS	↓ Cell viability<br><br>↑ Apoptosis	↑ p-ERK levels<br><br>↑ ROS<br><br>↓ JNK<br><br>↑ caspase-3 activation<br><br>↑ Phospho-p16^{INK4A}<br><br>↑ Phospho-p38<br><br>↑ Phospho-cyclin D1<br><br>ERα and ERβ mediated	[147]
LAPC-4	DES *	10^{-8} - 1 μM	96-well plates	72 h	MTT<br><br>Caspase 3 activity	↓ Cell viability<br><br>↑ Apoptosis	↓ p-ERK levels<br><br>↓ JNK	[147]

					ROS		↑ caspase-3 activation<br><br>↑ Phospho-$p16^{INK4A}$<br><br>↑ Phospho-p38<br><br>↑ Phospho-cyclin D1<br><br>ERβ and GPER mediated	
LNCaP	2-ME$_2$	5 μM	6-well plates<br><br>6 cm dishes	1 - 6 days	Cell count in haemocytometer<br><br>Flow cytometry<br><br>Annexin V-FITC and PI *in situ*<br><br>Caspase inhibition	↓ Cell growth<br><br>↑ Cytotoxicity	-	[208]
LNCaP	2-ME$_2$	10 μM	10 cm dishes	0 - 48 h	DNA fragmentation<br><br>TUNEL<br><br>Flow cytometry	↑ Apoptosis	SAPK/JNK activation<br><br>Bcl-2 phosphorylation	[213]
LNCaP	APVE$_2$*	0.0001 - 1 μM	75 cm^2 flasks<br><br>24- and 96-well plates	4 / 5 days	MTS<br><br>BrdUrd uptake<br><br>Flow cytometry<br><br>Caspase activity	↓ Cell number	-	[255]
LNCaP-FGC	2-ME$_2$	0.5 - 10 μM	96-well plates	4 - 72 h	MTS	↓ Cell growth	-	[235]

					Flow cytometry<br><br>Cyclin B1 and A-dependent kinase activity<br><br>Caspase-3 activity			
LNCaP-FGC	2-ME2	2 μM	-	24 h	Flow cytometry<br><br>Cyclin B1 and A-dependent kinase activity<br><br>Caspase-3 activity	↑ Cells in G1 phase<br><br>↓ Cells in S phase	↓ Cyclin A-dependent kinase activity<br><br>↑ p53<br><br>↑ p21	[235]
LNCaP-FGC	2-ME2	≥ 5 μM	-	24 h	Flow cytometry<br><br>Cyclin B1 and A-dependent kinase activity<br><br>Caspase-3 activity	↑ Cells in G2/M phase<br><br>↓ Cells in G1 phase<br><br>↑ Apoptosis	G2/M cell-cycle arrest<br><br>↑ Cyclin B1 and its associated kinase activity<br><br>↑ p53<br><br>↓ XIAP	[235]
LNCaP	2-ME2	5 and 10 μM	-	48 h	ELISA<br><br>Flow cytometry	↓ Cell number in G1 phase<br><br>↑ Cell number in S and G2/M phases<br><br>↑ Apoptosis	S-phase accumulation<br><br>G2/M arrest	[209]
LNCaP	E2<br>+<br>Cisplatin	0.1 and 0.2 μM<br>+<br>10, 50 and 100 μM	96-well plates<br><br>3.6 cm dishes	2 h	MTT	↓ Cell Viability	-	[265]

LNCaP	2-ME_2	1, 5 and 10 µM	75 cm^2 flasks	48 h	Western Blot	↑ β-catenin	-	[247]
LNCaP	2-ME_2	0.5, 1, 3 and 5 µM	96-well plates<br>6 cm dishes	24 and 72 h	MTS<br>TUNEL<br>Annexin-FITC<br>Colony formation		-	[212]
LNCaP	2-ME_2<br>+<br>Eugenol	0.5 µM<br>+<br>41 µg/mL	6 cm dishes	24 h	Apoptosis detection by morphological analysis<br>Annexin-FITC<br>Colony formation assay	↓ Cell growth	-	[212]
LNCaP	2-ME_2	0.01, 0.05, 0.1, 0.25,<br>0.5, 0.75 1 and 3 µM	96-well plates	72 h	Apoptosis detection	↑ Apoptosis	↑ cdc25c<br>↑ p34cdc2	[210]
LNCaP	HE3235 *<br>(in medium supplemented with AED)	0.05 µM	96-well plates<br>6-well plates	4 days	Cell counting in haemocytometer<br>Annexin V-FITC	↓ Proliferation<br>↑ Apoptosis	-	[263]
LNCaP	αE_2	0.01 - 5 µM	96-well plates	3 days	MTS<br>PSA measurement	↓ DHT-induced PSA<br>↓ DHT-induced cell growth	ER mediated	[176]
LNCaP	βE_2	0.01 - 5 µM	96-well plates	3 days	MTS<br>PSA measurement	↓ DHT-induced PSA	ER mediated	[176]

							↓ DHT-induced cell growth		
	LNCaP	3β-Adiol	0.01 µM	6-well plates<br>6 cm dishes	12, 24 and 48 h	TUNEL<br>Annexin V<br>Crystal violet<br>Immunocytochemistry	↑ Apoptosis<br>↓ Colony formation	↓ Bcl-2<br>↑ caspase-3/7 activity<br>↑ PUMA<br>↓ p53<br>↑ FOXO3a	[225]
	LNCaP	2-ME2	3 µM	-	24 h	Electrophoretic mobility shift assay (EMSA)<br>Chromatin immunoprecipitation (ChIP)	↓ c-FLIP promoter activity	-	[214]
	LNCaP	2-ME$_2$	0.5, 1, 2, 3 and 5 µM	96-well plates<br>6 cm dishes	24 and 48 h	Trypan blue exclusion<br>MTS<br>Flow cytometry<br>Mitotic index measurement<br>Light microscopy<br>TUNEL<br>FITC-PARP<br>Caspase activity	↓ Cell growth<br>↑ Cells in G2/M phase<br>↓ Cells in G1 phase<br>↓ Number of mitotic cells<br>↑ Apoptosis	G2/M arrest<br>↑ p21<br>↑ p34cdc2<br>↑ Wee 1 kinase<br>↑ phospho cdc2	[207]
	LNCaP	2-ME$_2$	3 µM		2 - 24 h	Real-Time PCR	↓ FLIP	-	[218]

LNCaP	2-ME2	1 µM	s	12 - 72 h	Annexin V-FITC/7-amino-actinomycin D<br><br>Caspase activity<br><br>Assessment of mitochondrial membrane potential	↑ Apoptosis	↑ Bcl-2 homology 3 (BH3)-only protein harakiri (Hrk)<br><br>↑ PUMA<br><br>↑ NOXA<br><br>↑ p-JNK<br><br>↑ c-Jun expression and phosphorylation<br><br>↑ Cytochrome C release<br><br>↑ caspase-3 activity	[246]
LN-AI	2-ME2	0.5 - 1 µM	96-well plates<br><br>6-well plates	24 - 72 h	MTS<br><br>Flow cytometry<br><br>DAPI staining<br><br>Clonogenic assay<br><br>Cyclin B1 and A-dependent kinase activity<br><br>Immunohistochemistry	↓ Cell growth<br><br>↑ Apoptosis	G2/M cell cycle arrest<br><br>↑ cleaved PARP<br><br>↑ p53<br><br>↑ p21	[211]
LN-AI	2-ME2<br>+<br>Docetaxel	0.5 - 1 µM<br>+<br>0.05 - 0.1 µM	96-well plates<br><br>6-well plates	24 - 72 h	MTS<br><br>Flow cytometry	↓ Cell growth<br><br>↑ Apoptosis	G2/M cell cycle arrest	[211]

					DAPI staining<br>Clonogenic assay<br>Cyclin B1 and A-dependent kinase activity<br>Immunohistochemistry	↓ Colonies formation	↑ cleaved PARP<br>↑ Cyclin B1-dependent kinase activity<br>↓ Cyclin A-dependent kinase activity<br>↑ p53<br>↑ p21	
LN-AI/CSS	2-ME_2	0.5 - 1 µM	96-well plates<br>6-well plates	24 - 72 h	MTS<br>Flow cytometry<br>DAPI staining<br>Clonogenic assay<br>Cyclin B1 and A-dependent kinase activity<br>Immunohistochemistry	↓ Cell growth<br>↑ Apoptosis	G2/M cell cycle arrest<br>↑ cleaved PARP	[211]
LN-AI/CSS	2-ME_2<br>+<br>Docetaxel	0.5 - 1 µM<br>+<br>0.05 - 0.1 µM	96-well plates<br>6-well plates	24 - 72 h	MTS<br>Flow cytometry<br>DAPI staining<br>Clonogenic assay	↓ Cell growth<br>↑ Apoptosis	G2/M cell cycle arrest<br>↑ cleaved PARP	[211]

					Cyclin B1 and A-dependent kinase activity<br><br>Immunohistochemistry			
22Rv1	E_2	1 µM	96-well plates	48 and 72 h	MTT<br><br>Flow cytometry	↓ Proliferation<br><br>↑ Apoptosis	↑ ERβ<br><br>↑ p75NTR<br><br>↓ NF-κB translocation to the nucleus	[201]
22Rv1	E_2<br>+<br>5-AzaC	1 µM<br>+<br>2 µg/ml	96-well plates	48 and 72 h	MTT<br><br>Flow cytometry	↓ Proliferation<br><br>↑ Apoptosis	↑ ERβ<br><br>↑ p75NTR<br><br>↓ NF-κB translocation to the nucleus	[201]
22Rv1	3β-Adiol	0.01 µM	6-well plates<br><br>6 cm dishes	12 and 24 h	Crystal violet	↓ Colony formation	↓ Bcl-2<br><br>↑ Bax<br><br>↑ PUMA<br><br>↓ p53<br><br>↑ FOXO3a	[225]
DU145	$2\text{-}ME_2$	5 µM	6-well plates<br><br>6 cm dishes	1 - 6 days	Cell count in haemocytometer<br><br>Flow cytometry<br><br>Annexin V-FITC and PI<br>*in situ*	↓ Cell growth<br><br>↑ Cytotoxicity<br><br>G2/M cell-cycle arrest<br><br>↑ Apoptosis	Caspase-mediated	[208]

					Caspase inhibition			
DU145	2-ME2	10 μM	10 cm dishes	0 - 48 h	DNA fragmentation assay<br>TUNEL<br>Flow cytometry	↑ Apoptosis	SAPK/JNK activation	[213]
DU145	APVE2 *	0.0001 - 1 μM	75 cm^2 flasks<br>24- and 96-well plates	4 / 5 days	MTS<br>BrdUrd uptake assay<br>Flow cytometry<br>Caspase activity	↓ Cell number<br>↑ Apoptosis	↑ caspase-3 activity	[255]
DU145	2-ME2	0.5 - 10 μM	96-well plates	4 - 72 h	MTS<br>Flow cytometry<br>Cyclin B1 and A-dependent kinase activity<br>Caspase-3 activity	↓ Cell growth	-	[235]
DU145	2-ME2	≥ 5 μM	-	24 h	Flow cytometry<br>Cyclin B1 and A-dependent kinase activity<br>Caspase-3 activity	↑ Cells in G2/M phase<br>↓ Cells in G1 phase<br>↑ Apoptosis	G2/M cell-cycle arrest<br>↑ Cyclin B1 and its associated kinase activity<br>↓ XIAP	[235]
DU145	2-ME2	5 and 10 μM	-	48 h	ELISA<br>Flow cytometry	↓ Cell number in G1 phase	S-phase accumulation	[209]

					Cyclin B1-cdc2 kinase activity	↑ Cell number in S and G2/M phases<br>↑ Apoptosis	G2/M arrest<br>↑ cyclin B1<br>↑ cdc2 phosphorylation<br>↑ wee1<br>↑ cdc25C<br>ERK 2 and JNK pathways	
DU145	2-ME_2	1, 5 and 10 μM	75 cm^2 flasks	48 h	Western Blot	↑ β-catenin<br>↓ β-catenin induced Cyclin D1	MEK-ERK 2 pathways<br>↓ Bcl-2	[247]
DU145	2-ME_2	0.5, 1, 3 and 5 μM	96-well plates<br>6 cm dishes	24 and 72 h	MTS<br>Apoptosis detection by morphological analysis<br>Annexin-FITC<br>Colony formation	No effect	-	[212]
DU145	2-ME_2<br>+<br>Eugenol	0.5 μM<br>+<br>41 μg/mL	6 cm dishes	24 h	Apoptosis detection by morphological analysis<br>Annexin-FITC<br>Colony formation	No effect	-	[212]

DU145	8β-VE_2 *	6 μM	multichambered slides	12 h	Immunohistochemistry	↑ Apoptosis	↑ caspase-3<br>↑ caspase-8	[253]
DU145	3β-Adiol	0.001 - 0.01 nM	10 mm dishes	48 h	Cell count in haemocytometer<br>Adhesion<br>Microchemotaxis<br>Matrigel gel	↓ Proliferation	-	[226]
DU145	2-ME_2	5 μM	-	24 h	Clonogenic cell survival	↑ Apoptosis<br>↓ Colony forming ability	↓ c-FLIP promoter activity	[214]
DU145	3β-Adiol	0.001 - 0.1 μM	48-well Boyden's microchemotaxis chambers	5 and 48 h	Chemotaxis<br>Western blot	↓ Migration	ERβ mediated<br>↑ E-cadherin	[227]
DU145	2-ME_2	0.5, 1, 2, 3 and 5 μM	96-well plates<br>6 cm dishes	24 and 48 h	Trypan blue exclusion<br>MTS<br>Flow cytometry<br>Mitotic index measurement<br>Light microscopy<br>TUNEL<br>FITC-PARP	↓ Cell growth<br>↑ Cells in G2/M phase<br>↓ Cells in G1 phase<br>↓ Number of mitotic cells<br>↑ Apoptosis	G2/M arrest	[207]

					Caspase activity			
DU145	2-ME2	1 μM		12 - 72 h	Annexin V-FITC/7-amino-actinomycin D	↑ Apoptosis		[246]
PC3	2-ME2	5 μM	6-well plates<br><br>6 cm dishes	1 - 6 days	Cell count in haemocytometer<br><br>Flow cytometry<br><br>Annexin V-FITC and PI *in situ*<br><br>Caspase inhibition	↓ Cell growth<br><br>↑ Cytotoxicity	-	[208]
PC3	2-ME2	10 μM	10 cm dishes	0 - 48 h	DNA fragmentation<br><br>TUNEL<br><br>Flow cytometry	↑ Apoptosis	SAPK/JNK activation<br><br>Bcl-2 phosphorylation	[213]
PC3	2-ME2	1 - 1.5 μg/mL<br><br>(~ 3 – 5 μM)	6-well plates	0 - 48h	Immunoblotting<br><br>Flow Cytometry<br><br>Electrophoretic mobility shift	↑ Apoptosis	G2/M cell-cycle arrest<br><br>Fas-mediated<br><br>↓ NF-kβ/FLIP signaling<br><br>↑ DISC formation<br><br>Akt inhibition<br><br>ERK-mediated	[215]
PC3	2-ME2	1, 3, 5, 10, and 20 μM	24-well plates	1 - 12 days	Multicellular aggregate culture	↓ Cell aggregates growth	-	[216]

					DAPI staining<br><br>TUNEL	↑ Apoptosis		
PC3	APVE2 *	0.0001 - 1 μM	75 cm^2 flasks<br><br>24- and 96-well plates	4 / 5 days	MTS<br><br>BrdUrd uptake assay<br><br>Flow cytometry<br><br>Caspase activity	↓ Cell number	G2/M cell-cycle arrest	[255]
PC3	2-ME2	0.5 - 10 μM	96-well plates	4 - 72 h	MTS<br><br>Flow cytometry<br><br>Cyclin B1 and A-dependent kinase activity<br><br>Caspase-3 activity	↓ Cell growth	-	[235]
PC3	2-ME2	≥ 5 μM	-	24 h	Flow cytometry<br><br>Cyclin B1 and A-dependent kinase activity<br><br>Caspase-3 activity	↑ Cells in G2/M phase<br><br>↓ Cells in G1 phase	↑ cyclin B1 and its associated kinase activity	[235]
PC3	2-ME2	10 μM	-	24 h	Caspase-3 activity	↑ Apoptosis	G2/M cell-cycle arrest<br><br>↓ XIAP	[235]
PC-3U	2-ME2	10 μM	10 cm dishes	12 h	DAPI staining<br><br>M30 staining	↑ Apoptosis<br><br>↑ c-Myc expression	Smad7 expression	[217]

						↑ β-catenin stabilization	p38 MAPK and JNK pathways	
PC3	2-ME_2	5 and 10 μM	-	48 h	ELISA<br><br>Flow cytometry<br><br>Cyclin B1-cdc2 kinase activity	↓ Cell number in G1 phase<br><br>↑ Cell number in S and G2/M phases<br><br>↑ Apoptosis	S-phase accumulation<br><br>G2/M arrest	[209]
PC3	E_2	0.001, 0.01 and 0.1 μM	96-well plates	24, 48, 72 and 96 h	MTT<br><br>Acridine orange and ethidium bromide staining<br><br>Flow cytometry	↓ Cell Viability<br><br>↓ Proliferation<br><br>↑ Apoptosis	↓ IGF-1R<br><br>↑ IGFBP-3<br><br>↑ IGFBP-4<br><br>↓ MMP-2<br><br>↓ MMP-9	[199]
PC3	E_2<br>+<br>Cisplatin	0.1 and 0.2 μM<br>+<br>10, 50 and 100 μM	96-well plates<br><br>3.6 cm dishes	2 h	MTT assay	↓ Cell Viability	-	[265]
PC3	2-ME_2	1, 5 and 10 μM	75 cm^2 flasks	48 h	Western Blot	↑ β-catenin	-	[247]
PC3	2-ME_2	0.5, 1, 3 and 5 μM	96-well plates<br><br>6 cm dishes	24 and 72 h	MTS<br><br>Apoptosis detection by morphological analysis	↓ Cell growth	-	[212]

					Annexin-FITC<br><br>Colony formation assay			
PC3	2-ME2<br>+<br>Eugenol	0.5 µM<br>+<br>41 µg/mL	6 cm dishes	24 h	Apoptosis detection by morphological analysis<br><br>Annexin-FITC<br><br>Colony formation assay	↓ Cell growth<br><br>↑ G2/M population<br><br>↓ G1 population<br><br>↑ Apoptosis	↓ Bcl-2<br><br>↑ Bax<br><br>↓ MMP	[212]
PC3	8β-VE2*	6 µM	multichambered slides	12 h	Immunohistochemistry	↑ Apoptosis	↑ caspase-3<br><br>↑ caspase-8	[253]
PC3	2-ME2	0.5 - 1 µM	96-well plates<br><br>6-well plates	24 - 72 h	MTS<br><br>Flow cytometry<br><br>DAPI staining<br><br>Clonogenic assay<br><br>Cyclin B1 and A-dependent kinase activity<br><br>Immunohistochemistry	↓ Cell growth	G2/M cell cycle arrest<br><br>↑ p21	[211]
PC3	2-ME2<br>+<br>Docetaxel	0.5 - 1 µM<br>+<br>0.05 - 0.1 µM	96-well plates<br><br>6-well plates	24 - 72 h	MTS<br><br>Flow cytometry<br><br>DAPI staining<br><br>Clonogenic assay	↓ Cell growth<br><br>↓ Colonies formation	G2/M cell cycle arrest<br><br>↑<br>B1-dependent kinase activity<br><br>↑ p21	[211]

					Cyclin B1 and A-dependent kinase activity<br>Immunohistochemistry			
PC3/AR	2-ME$_2$	0.5 - 1 μM	96-well plates<br>6-well plates	24 - 72 h	MTS<br>Flow cytometry<br>DAPI staining<br>Clonogenic assay<br>Cyclin B1 and A-dependent kinase activity<br>Immunohistochemistry	↓ Cell growth	G2/M cell cycle arrest	[211]
PC3/AR	2-ME$_2$<br>+<br>Docetaxel	0.5 - 1 μM<br>+<br>0.05 - 0.1 μM	96-well plates<br>6-well plates	24 - 72 h	MTS<br>Flow cytometry<br>DAPI staining<br>Clonogenic assay<br>Cyclin B1 and A-dependent kinase activity<br>Immunohistochemistry	↓ Cell growth	G2/M cell cycle arrest	[211]
PC3	3β-Adiol	0.001 – 0.01 μM	10 mm dishes	48 h	Cell count in haemocytometer	↓ Proliferation	-	[226]

PC3	3β-Adiol	0.01 μM	6-well plates<br>6 cm dishes	12 and 24 h	Crystal violet	↓ Colony formation	↓ Bcl-2<br>↑ cleaved caspase-9<br>↑ PUMA<br>↑ FOXO3a	[225]
PC3	8β-VE2*	0.01 μM	6-well plates<br>6 cm dishes	12 and 24 h	Crystal violet	↓ Colony formation	↓ Bcl-2<br>↑ cleaved caspase-9<br>↑ FOXO3a	[225]
PC3-Luc	3β-Adiol	0.001 – 0.01 μM	10 mm dishes	48 h	Cell count in haemocytometer<br>Thymidine incorporation assays<br>Adhesion<br>Microchemotaxis<br>Matrigel gel	↓ Proliferation<br>↑ Cell adhesion<br>↓ Migration<br>↓ Invasion	ERβ mediated	[226]
PC3	E_2	10^{-8} - 1 μM	96-well plates	72 h	MTT<br>Caspase-3 activity<br>Necroptosis<br>ROS	↓ Cell viability<br>↑ ROS	↑ p-ERK levels<br>↑ ROS<br>↑ JNK<br>↑ Phospho-$p16^{INK4A}$<br>↑ Phospho-p38	[147]

							↑ Phospho-cyclin D1<br><br>ERβ and GPER mediated	
PC3	DES*	10^{-8} - 1 μM	96-well plates	72 h	MTT<br><br>Caspase-3 activity<br><br>Necroptosis<br><br>ROS		↓ p-ERK levels<br><br>↓ JNK<br><br>↑ Phospho-$p16^{INK4A}$<br><br>↑ Phospho-p38<br><br>↓ Phospho-cyclin D1	[147]
PC3	2-ME_2	3 μM		24 h	EMSA<br><br>ChIP	-	↓ c-FLIP promoter activity	[214]
PC3	3β-Adiol	0.001 – 0.1 μM	48-well Boyden's microchemotaxis chambers	5 and 48 h	Chemotaxis<br><br>Western blot	↓ Migration	-	[227]
PC3-AR	3β-Adiol	0.001 – 0.1 μM	48-well Boyden's microchemotaxis chambers	5 and 48 h	Chemotaxis<br><br>Western blot	↓ Migration	-	[227]
PC3	2-ME_2	3 μM		2 - 24 h	Real-Time PCR<br><br>Immunoblotting<br><br>Immunoprecipitation	↑ Apoptosis	Cleavage of Bid<br><br>caspase-8 activation<br><br>↓ FLIP<br><br>↓ Bcl-2	[218]
PC3	2-ME_2	1 μM		12 - 72 h	Annexin V-FITC/7-amino-actinomycin D<br><br>Caspase activity	↑ Apoptosis	↑ Bcl-2 homology 3 (BH3)-only protein harakiri (Hrk)	[246]

					Assessment of mitochondrial membrane potential		↑ PUMA<br>↑ NOXA<br>↑ Bim<br>↑ p-JNK<br>↑ c-Jun expression and phosphorylation<br>↑ cytochrome C release<br>↑ caspase-3 activity	
VCaP	8β-VE2*	5 or 25 μM	96-well plates	24, 48, and 72 h	Measurement of cell survival and apoptosis induction	↓ Cell survival<br>↑ Apoptosis	↓ AR	[252]
VCaP rev	8β-VE2*	5 or 25 μM	96-well plates	24, 48, and 72 h	Measurement of cell survival and apoptosis induction	↓ Cell survival<br>↑ Apoptosis	↑ AR	[252]
VCaP AA	8β-VE2*	5 or 25 μM	96-well plates	24, 48, and 72 h	Measurement of cell survival and apoptosis induction	↓ Cell survival<br>↑ Apoptosis	↓ AR	[252]
C4-2B	HE3235*	0.01 and 0.05 μM	6-well plates	3 days	Trypan blue exclusion	↓ Proliferation	-	[264]
MDA-Pca 2b	αE2	0.01 - 5 μM	96-well plates	3 days	MTS<br>PSA measurement	↓ Cortisol-induced PSA	ER mediated	[176]
MDA-Pca 2b	βE2	0.01 - 5 μM	96-well plates	3 days	MTS<br>PSA measurement	↓ Cortisol-induced PSA	ER mediated	[176]

	Human primary epithelial cell cultures from benign regions of prostate tissue	8β-VE2*	6 µM			Flow cytometry	↑ Apoptosis	-	[254]
	Primary culture from prostatic epithelial cells of C57BL6/J wild type mice	8β-VE2*	6 µM	12-well plates	7 days	Spheroid assay	↓ Self-renewal ability	-	[254]
in vivo	Wistar (*Rattus norvegicus*) rats	E_2	250 mg/ kg/ day	Intraperitoneal injection	5 days	Prostate weight measure<br><br>Ki-67-Fluorescent immunohistochemistry<br><br>TUNEL<br><br>Caspase-3 activity	↓ Prostate weight<br><br>↓ Proliferation<br><br>↑ Apoptosis<br><br>↓ SFC/c-KIT expression	↑ Fas and Fas L<br><br>↑ caspase-8<br><br>↑ caspase-3 activity	[202]
	Copenhagen Fisher rats + Dunning R3327-PAP tumors	β-estradiol 3-benzoate*	50 µg	s.c. injections	12, 24, 72 and 168 h	Tumor volume measurement<br><br>PCNA-immunohistochemistry<br><br>*In situ* detection of apoptotic cells	↓ Tumor volume<br><br>↓ Proliferation<br><br>↑ Apoptosis	-	[206]
	Copenhagen Fisher rats + Dunning R3327-PAP tumors	E_2	50 µg / day	s.c. injections	6 weeks	Volume measurement and morphological examination of tumors	↓ Tumor growth<br><br>Tumor degeneration	-	[204]

Copenhagen Fisher rats + Dunning R3327-PAP tumors	E_2	50 µg / day	s.c. injections	4, 12 and 24 h or 6 weeks	Morphological examination<br>Immunohistochemistry<br>TUNEL	↑ Apoptosis	↑ TGF-β1<br>↑ TGF-β RI<br>↑ TGF-β RII	[205]
Copenhagen Fisher rats + Dunning R3327-PAP tumors	2-ME_2	12.5 mg / kg / day	Intraperitoneal injection	14 days	Measure of tumor diameter<br>TUNEL	↓ Tumor growth<br>↑ Apoptosis	-	[213]
Gγ/T-15 transgenic mouse	2-ME_2	75 mg / kg	s.c. implanted slow-release tablets	21 days	Palpation	↓ Tumor weight	-	[208]
HONDA xenograft tumor model in nu/nu mice	2-ME_2	20 mg / kg per day	Intraperitoneal injection	72 h	TUNEL<br>CD31 immunohistochemical analysis	↓ Tumor growth<br>↑ Apoptosis<br>↓ Microvessel density	-	[219]
LAPC-4 xenograft tumor model in SCID mice	αE_2	2 mg plus 18 mg cholesterol	s.c. implanted pellet	4 weeks	PSA and testosterone levels determination	↓ Tumor growth	-	[200]
LAPC-4 xenograft tumor model in SCID mice	βE_2	2 mg plus 18 mg cholesterol	s.c. implanted pellet	4 weeks	PSA and testosterone levels determination	↓ PSA levels	-	[200]
PC3 tumor model in nu/nu mice	2-ME_2	20 mg / kg per day	Intraperitoneal injection	72 h	TUNEL<br>CD31 immunohistochemical analysis	↑ Apoptosis<br>↓ Microvessel density	-	[219]

LNCaP tumor model in nude athymic mice	2-ME$_2$	25 mg / kg and 75 mg / kg per day	Orally in diet or water	62 days	Tumor histology<br>TUNEL<br>Determination of serum PSA	↓ Tumor size<br>↓ Necrosis<br>↑ Apoptosis<br>↓ PSA levels	-	[210]
LuCaP35V tumor model in male CB-17 SCID mice	HE3235*	160 mg / kg	Intraperitoneal injection	5 days / week for 4 weeks	Determinations of intratumoral androgens levels<br>PSA determination<br>Immunohistochemistry	↓ Tumor growth<br>↑ PSA levels<br>↓ Testosterone and DHT levels<br>↓ AR expression	-	[264]
C4-2B tumor model in male CB-17 SCID mice	HE3235*	160 mg / kg	Intraperitoneal injection	7 days / week for 4 weeks	Radiography<br>Bone mineral density measurements<br>Immunohistochemistry	↓ Weight of tumor tibiae<br>↓ PSA levels<br>↓ AR expression	-	[264]
Balb-c/Nude mice	8β-VE$_2$*	300 µg / kg / day	s.c. injection	3 days	Immunohistochemistry	↑ Apoptosis	↑ caspase-8<br>Extrinsic pathway<br>TNFα mediated	[253]
BPH tissue xenografts in NOD/SCID mice	8β-VE$_2$*	300 µg / kg / day	s.c. injection	3 days	Immunohistochemistry	↑ Apoptosis	↑ caspase-8<br>Extrinsic pathway<br>TNFα mediated	[253]
PCa tissue xenograft in	8β-VE$_2$*	300 µg / kg / day	s.c. injection	3 days	Immunohistochemistry	↑ Apoptosis	↑ caspase-8	[253]

NOD/SCID mice							Extrinsic pathway	
FG/Tag transgenic mouse model of AI-PC	2-ME2	150 mg / kg	-	24 and 48 h	Flow cytometry<br>Immunohistochemistry	↓ Primary prostate tumors<br>↑ Apoptosis<br>↓ Cell proliferation↓ Angiogenesis	G2/M cell cycle arrest<br>↑ Cleaved caspase-3	[211]
FG/Tag transgenic mouse model of AI-PC	2-ME2 + Docetaxel	75 mg / kg + 2.5 mg / kg	-	24 and 48 h	Flow cytometry<br>Immunohistochemistry	↓ Primary prostate tumors<br>↑ Apoptosis<br>↓ Cell proliferation<br>↓ Angiogenesis	G2/M cell cycle arrest<br>↑ cleaved caspase-3	[211]
FG/Tag transgenic mouse model of AI-PC	2-ME2 + Docetaxel	150 mg / kg + 5 mg / kg	-	24 and 48 h	Flow cytometry<br>Immunohistochemistry	↓ Primary prostate tumors<br>↑ Apoptosis<br>↓ Cell proliferation<br>↓ Angiogenesis	G2/M cell cycle arrest<br>↑ cleaved caspase-3	[211]
AED-stimulated LNCaP tumor model in castrated SCID mice	HE3235*	200 ml / day	Intraperitoneal injection	21 days	-	↓ Tumor incidence<br>↓ Tumor growth	-	[263]
PC3 xenograft tumor model in	3β-Adiol	2.5 mg / kg / day	s.c. injection	3 weeks	BLI acquisition	↓ Proliferation of established	-	[226]

BALB/c nu/nu nude mice						tumor		
PC3 xenograft tumor model in BALB/c nu/nu nude mice	3β-Adiol	2.5 mg / kg / day	s.c. implanted pellet	4 weeks	BLI acquisition	↓ PCa progression<br>↓ Metastasis	-	[226]
LuCaP 35 xenograft tumor model in SCID mice	E_2	0.36 mg	s.c. implantation of slow-release Trocar pellets	90 days	Measurement of tumor volume	↓ Tumor growth<br>↑ Survival<br>↓ Testosterone and DHT levels	-	[133]
LNCaP xenograft tumor model in nude mice (Harlan™)	αE_2	2 mg αE_2 plus 18 mg cholesterol	s.c. implanted pellet	4 weeks	Measurement of tumor size	↓ tumor growth<br>↓ Tumor size	-	[176]
LNCaP xenograft tumor model in nude mice (Harlan™)	βE_2	2 mg βE_2 plus 18 mg cholesterol	s.c. implanted pellet	4 weeks	Measurement of tumor size	↓ Tumor size	-	[176]
C57BL6/J wild type mice	8β-VE_2*	6 μM		24 h	Flow cytometry<br>Immunohistochemistry	↑ Apoptosis	-	[254]
LNCaP xenograft animal models	αE_2	2 mg αE_2 plus 18 mg cholesterol	s.c. implanted pellet	4 weeks	Immunohistochemistry	↓ Microvessel number	-	[278]
LNCaP xenograft animal models	βE_2	2 mg βE_2 plus 18 mg cholesterol	s.c. implanted pellet	4 weeks	Immunohistochemistry	↓ Microvessel number	-	[278]
LAPC-4 xenograft animal models	αE_2	2 mg αE_2 plus 18 mg cholesterol	s.c. implanted pellet	4 weeks	Immunohistochemistry	↓ Microvessel number	-	[278]

LAPC-4 xenograft animal models	βE_2	2 mg βE_2 plus 18 mg cholesterol	s.c. implanted pellet	4 weeks	Immunohistochemistry	↓ Microvessel number	-	[278]
LNCaP xenograft tumor model in BALB/c nude mice	2-ME_2	150 mg / kg	Intraperitoneal injection	4 weeks	Measurement of tumor size<br>Immunohistochemistry	↓ Tumor growth<br>↓ Proliferation<br>↑ Apoptosis<br>↓ Microvessel density	↑ Bax<br>↓ Bcl-2<br>caspase-3 mediated<br>↓ pAkt<br>↓ VEGF	[220]
LNCaP xenograft tumor model in BALB/c nude mice	2-ME_2 + Quercetin	150 mg / kg + 75 mg / kg	Intraperitoneal injection	4 weeks	Measurement of tumor size<br>Immunohistochemistry	↓ Tumor growth<br>↓ Proliferation<br>↑ Apoptosis<br>↓ Microvessel density	↑ Bax<br>↓ Bcl-2<br>caspase-3 mediated<br>↓ pAkt<br>↓ VEGF	[220]
PC3 xenograft tumor model in BALB/c nude mice	2-ME_2	150 mg / kg	Intraperitoneal injection	4 weeks	Measurement of tumor size<br>Immunohistochemistry	↓ Tumor growth<br>↓ Proliferation<br>↑ Apoptosis<br>↓ Microvessel density	↑ Bax<br>↓ Bcl-2<br>caspase-3 mediated<br>↓ pAkt<br>↓ VEGF	[220]
PC3 xenograft tumor model in BALB/c nude mice	2-ME_2 + Quercetin	150 mg / kg + 75 mg / kg	Intraperitoneal injection	4 weeks	Measurement of tumor size<br>Immunohistochemistry	↓ Tumor growth<br>↓ Proliferation<br>↑ Apoptosis	↑ Bax<br>↓ Bcl-2<br>caspase-3 mediated	[220]

						↓ Microvessel density	↓ pAkt<br>↓ VEGF	
DU145 xenograft tumor model in BALB/ cA-nu castrated mice	E_2	3.4 mg	s.c implanted pellets	25 - 35 days	Measurement of tumor size<br>TUNEL<br>Soft-agar colony formation<br>Immunohistochemistry<br>Matrigel plus angiogenesis	↓ Tumor growth<br>↓ Angiogenesis<br>↓ Apoptosis	↓ FOXO1<br>ERβ and PDGFA, KLF5 pathway	[14]
PC3 xenograft tumor model in BALB/ cA-nu castrated mice	E_2		s.c implanted pellets	25 - 35 days	Measurement of tumor size<br>TUNEL<br>Soft-agar colony formation<br>Immunohistochemistry<br>Matrigel plus angiogenesis	↓ Tumor growth<br>↓ Angiogenesis<br>↓ Apoptosis	↓ FOXO1<br>ERβ and PDGFA, KLF5 pathway	[14]
TRAMP mice in pure C57BL/6 8-week-old (hyperplasia)	2-ME_2	50 mg / kg	Orally in diet	16 weeks	Measurement of prostate weight<br>Histopathologic evaluation<br>Analysis of hormone levels in serum	↓ Prostate weight<br>↓ Neoplastic Progression<br>↓ Testosterone levels	↑ TSG-6	[221]

					Immunochemistry<br><br>Invasion			
TRAMP mice in pure C57BL/6 24-week-old (metastasis)	2-ME_2	50 mg / kg	Orally in diet	8 weeks	Measurement of prostate weight<br><br>Histopathologic evaluation<br><br>Analysis of hormone levels in serum<br><br>Immunochemistry<br><br>Invasion	↓ Prostate weight<br><br>↓ Carcinogenesis<br><br>↓ Testosterone levels	↑ TSG-6	[221]
LuCaP 35 xenograft tumor in 15 ovariectomized BALB/c nu/nu mice	E_2	-	s.c. implanted pellets	90 days	Measurement of tumor volume	↓ Tumor volume	-	[203]
TRAMP mice (22-25 weeks)	2-ME_2	50 mg / kg	Orally in water	25 weeks	Palpable tumors number<br><br>Volume of the prostate seminal vesicle complex<br><br>Histological evaluation	Tumor regression<br><br>↓ Palpable tumor occurrence<br><br>↓ Prostate weight<br><br>↓ Proliferation<br><br>↓ Apoptosis	↓ Sp1<br><br>↓ FLIP	[218]

							(restoring normal tissue architecture)		
**Patients**	Prostate Carcinoma	Polyestradiol phosphate*	240 mg	Intramuscular injections	2 years	Radioimmunological or immunoradiometric assays	↓ Bone *turnover*	↓ Osteocalcin<br>↓ PICP<br>↓ PIIINP<br>↓ ICTP	[127]

* - synthetic compound
