# Supplementary material for: The Pros and Cons of Estrogens in Prostate Cancer: An Update with a Focus on Phytoestrogens
Source: Biomedicines. 2024 Jul 23;12(8):1636. doi: 10.3390/biomedicines12081636 (PMC11351860; doi:10.3390/biomedicines12081636)
Supplement: Supplementary file 1 [file biomedicines-12-01636-s001.zip › Supplementary Table S2.pdf]

**Supplementary Table S2.** Phytoestrogens with a role in PCa

| Phytoestrogens | Class | Subclass | Compound | Source | Cell line/ Animal Model | Concentration/ Dose | Effect | Activated Pathway | Ref. |
|---|---|---|---|---|---|---|---|---|---|
| **Phenolic compounds** | **Flavonoids** | **Flavones** | **Apigenin** | Fruits<br><br>Vegetables | LNCaP<br><br>DU145<br><br>PC3<br><br>22RV1<br><br>PCa stem cells (PCSCs)<br><br>MAT-Lylu<br><br>TRAMP mice<br><br>PC3 xenograft<br><br>C57BL/TGN TRAMP mice | 0 – 100 µM (cell lines)<br><br>Up to 50 µg/day (animals) | ↓ Cell viability<br><br>↓ Proliferation<br><br>Cell cycle arrest at G2/M phase<br><br>Cell cycle arrest at G1 phase<br><br>↑ Apoptosis<br><br>↓ Hypoxia<br><br>↓ Angiogenesis<br><br>↓ Cell motility<br><br>↓ Migration<br><br>↓ Invasion<br><br>↓ PCa progression<br><br>Reversion of EMT | ↓ Cyclin D1, D2 and E<br><br>↓ CDK2, 4 and 6<br><br>↑ p53<br><br>↑ p21<br><br>↑ p27<br><br>↓ pRb<br><br>↑ ROS<br><br>↑ Bax/Bcl-2 ratio<br><br>↑ TRAIL (DR5) receptors<br><br>↑ Caspase-8, 9, 10 and 3<br><br>↑ Cytochrome c | [301,311–313,315–325,327–340,344,346,348–350] |

| | | | | | | | | | |
|---|---|---|---|---|---|---|---|---|---|
| | | | | | | | Modulation of glucose and lipid metabolism<br><br>↑ Survival | ↓ β-catenin<br><br>↓ IGF axis<br><br>↓ HIF$\alpha$<br><br>↓ VEGF<br><br>GLUT1 modulation<br><br>↓ FASN<br><br>↓ Akt/ PI3K<br><br>↑ MAPK<br><br>↓ NFκB<br><br>↑ PTEN<br><br>↓ Snail<br><br>↓ MMPs 1, 7 and 9<br><br>↓ AR<br><br>↑ ERβ | |
| | | | **Chrysin** | Honey<br><br>Propolis | LNCaP<br><br>C4-2, | 0 – 100 μM | ↓ Proliferation<br><br>↓ 3D cell growth | ↑ TRAIL receptors | [311,352,362,363,365] |

| | | | | | | | | | |
|---|---|---|---|---|---|---|---|---|---|
| | | | | Blue passion flowers | DU145<br><br>PC3<br><br>PC3 3D cell culture<br><br>PC-3 Xenograft in Nude Mice | | ↑ Apoptosis<br><br>↓Vasculogenic mimicry Tube-Formation<br><br>↓ tumor growth | ↓ Bcl-2<br><br>↑ Caspase-3<br><br>↓ CDK6<br><br>↓ Cyclin D1<br><br>↑ ROS<br><br>↓ HIF$\alpha$<br><br>↓ SPHK<br><br>↓ VEGF<br><br>↓ Akt/PI3K<br><br>↓ VE-cadherin<br><br>↓ PCNA. | |
| | | **Isoflavones** | **Biochanin A** | Red clover<br><br>Soy<br><br>Alfalfa sprouts<br><br>Peanuts<br><br>Legumes | LNCaP<br><br>DU145<br><br>PC3<br><br>LNCaP xenografts | 0 – 100 μM | ↓ Proliferation<br><br>Cell cycle arrest at G0/G1 phase<br><br>Cell cycle arrest at G2/M phase<br><br>↑ Apoptosis<br><br>↓ PSA | ↓ Cyclin B and E<br><br>↓ c-Myc<br><br>↑ p21<br><br>↑ Bax<br><br>↓ Bcl-2<br><br>↑ TRAIL receptors | [319,368,370–372] |

| | | | | | | | | | |
|---|---|---|---|---|---|---|---|---|---|
| | | | | | | | | ↑ DNA damage<br><br>↓ pAkt<br><br>↓ pERK<br><br>↑ ERβ | |
| | | | **Daidzein** | Soybean<br><br>Other legumes and fruits | LNCaP<br><br>DU145<br><br>PC3<br><br>Wistar unilever rat<br><br>F344 rat<br><br>R3327 rat | 0 – 200 μM<br><br>Up to 2000 mg/kg or 200 ng/L | ↓ Cell viability<br><br>↓ Proliferation<br><br>Cell cycle arrest at G2/M phase<br><br>↑ Apoptosis<br><br>↓ Angiogenesis<br><br>↓ Migration<br><br>↓ Invasion<br><br>↓ PCa progression<br><br>Modulation of glucose and lipid metabolism<br><br>↓ PSA levels<br><br>↑ PSA level free/total PSA ratio | ↓ Cyclin A and B1<br><br>↑ p53<br><br>↑ p21<br><br>↑ Bax<br><br>↑ TRAIL (DR4 and DR5) receptors<br><br>↓ MMP-13<br><br>↓ HIF*α* | [374–385,390,446] |

| | | | | | | | | | |
|---|---|---|---|---|---|---|---|---|---|
| | | | **Equol** | Daidzein's metabolite | LNCaP<br><br>DU145<br><br>PC3<br><br>Dunning R3327 Prostate Adenocarcinoma in Copenhagen × Fisher F1 male rats<br><br>TRAMP mice<br><br>Human Patients<br><br>Male Long-Evans rats | 0-100 μM | ↓ Proliferation<br><br>↓ Growth<br><br>Cell cycle arrest at G0 phase<br><br>↑ Apoptosis<br><br>↓ Migration<br><br>↓ Invasion<br><br>↓ PSA | ↑ TRAIL receptors<br><br>↑ DNA damage<br><br>↓ MMP-2 and MMP-9<br><br>↑ Antioxidant defenses<br><br>↑ Degradation of AR<br><br>Affinity to ERβ | [377,391,397,409,413–421] [358,422,423] |
| | | | **Formononetin** | Red clover plants | LNCaP<br><br>DU145<br><br>PC3 | 0 – 100 μM | Cell cycle arrest at G0/G1 phase<br><br>↑ Apoptosis | ↓ Cyclin D1<br><br>↓ CDK4<br><br>↑ Bax/Bcl-2 ratio<br><br>↓ pAkt<br><br>↓ ERK1/2<br><br>↓ IGF-1/IGF-1R | [425–430] |
| | | | **Genistein** | Soybeans<br><br>Peas | LNCaP<br><br>DU145 | 0 – 200 μM<br><br>25 mg/kg to 1g/kg | ↓ Cell viability<br><br>↓ Proliferation | ↓ Cyclin B<br><br>↑ p21 | [380,383,394,438–443,449,452– |

| | | | | | | | | | |
|---|---|---|---|---|---|---|---|---|---|
| | | | | Lentils<br>Beans | PC3<br>3D culture of PC3 cells<br>MAT-LyLu<br>LTL1630a<br>BALB/c nu/nu nude mice<br>C57BL/6 mice<br>BALB/c mice<br>Lobund-wistar rat<br>C17 beige SCID mice<br>Sprague dawley rat<br>SV-40 TAG rat<br>ACI/Seg rat<br>TRAMP mice<br>SCID mice<br>Xenograft tumor mouse model established with 22RV1 cells | | Cell cycle arrest at G2/M phase<br>↑ Apoptosis<br>↑ Angiogenesis<br>↓ Migration<br>↓ Invasion<br>Reversion of EMT<br>↓ PSA<br>↓ Advanced PCa incidence<br>↓ Tumor growth rate<br>↓ Tumor weight<br>↑ Survival | ↑ p53<br>↑ ROS<br>↑ GSH<br>↑ Catalase<br>↓ NO<br>↓ MMP-2<br>↑ E-cadherin<br>↓ Vimentin<br>↓ FAK<br>↓ VEGF<br>↓ COX-2 pathway<br>↓ GLUT1<br>↓ pAkt<br>↓ EGF-induced activation of Akt | 461,463–466,511]<br>[468]<br>[358,364,445–450,458,515] |

| | | | | | | | | | |
|---|---|---|---|---|---|---|---|---|---|
| | | | | | | | | ↓ p38 MAPK<br><br>↓ NFkβ activation<br><br>Hedgehog pathway<br><br>↑ERβ<br><br>↓ AR<br><br>↑ PTEN<br><br>↓ IGF-1<br><br>↓ 5α-reductase-2<br><br>↓ GSK-3β phosphorilation<br><br>↓ Cyclin D1<br><br>↓ Osteopontin<br><br>↓ SRC-3 | |
| | | **Flavanones** | **Naringenin** | Grapefruit<br><br>Orange | LNCaP<br><br>PC3<br><br>22RV1 | 0 – 75 μM | ↓ Proliferation<br><br>↑ Apoptosis<br><br>↓ Migration | ↑ Bax<br><br>↓ Bcl-2<br><br>↑ E-cadherin | [520–523] |

| | | | | | | | | | |
|---|---|---|---|---|---|---|---|---|---|
| | | | | | MAT-LyLu | | ↓ Invasion<br>↓ Metastasis | ↓ Vimentin<br>↓ MAPK<br>↑ SOD activity<br>↓ Glutathione reductase<br>↑ Lipid peroxidation | |
| | | **Flavonols** | **Kaempferol** | Kale<br>Beans<br>Tea<br>Spinach<br>Broccoli | LNCaP<br>22Rv1<br>DU145<br>PC3 | 0 – 1000 μM | ↓ Proliferation<br>Cell cycle arrest at G1 phase<br>Cell cycle arrest at S phase<br>Cell cycle arrest at G2/M phase<br>↑ Apoptosis | ↑ TRAIL receptors<br>↑ Caspase-8, 9 and 3<br>↓ Akt<br>↑ ERK<br>↓ NFkβ<br>↓ IGF-I<br>↓ Ki67 | [381,527,530,532–536,558] |
| | | | **Myricetin** | Vegetables<br>Fruits<br>Nuts<br>Berries | PC3<br>C4-2B<br>C4-2B Xenograft | 0 – 300 μM | ↓ Proliferation<br>↑ Apoptosis<br>↓ Tumor growth<br>↓ PCa progression | ↓ CXCR4 | [543,544,551] |

| | | | | | | | | | |
|---|---|---|---|---|---|---|---|---|---|
| | | | | Tea<br><br>Red wine | | | | | |
| | | | **Quercetin** | Capers<br><br>Lovage<br><br>Dill<br><br>Cilantro<br><br>Onions<br><br>Apples<br><br>Berries | LNCaP<br><br>LNCaP/R (resistant to docetaxel)<br><br>DU145<br><br>PC3<br><br>PC3/R (resistant to docetaxel)<br><br>SCIV mice<br><br>Sprague dawley rat<br><br>BALB/c nude mice<br><br>TRAMP mice | 0 - 160 µM<br><br>Up to 200 mg/kg or 400 mg/day | ↓ Cell viability<br><br>↓ Proliferation<br><br>Cell cycle arrest at S phase<br><br>Cell cycle arrest at sub-G0/G1 phase<br><br>↑ Apoptosis<br><br>↓ Angiogenesis<br><br>↓ Migration<br><br>↓ Invasion<br><br>↓ EMT | ↓ Cyclin B<br><br>↓ CDK1<br><br>↓ pRb<br><br>↑ p21<br><br>↓ Bid and Bcl-2<br><br>↑ Bax<br><br>↑ TRAIL and DR5 receptors<br><br>↓ Heat shock protein 90<br><br>↑ Caspase-8, 9 and 3<br><br>↑ Autophagy<br><br>↑ ROS<br><br>↓ MMP-2 and MMP-9 | [42,43,358,533, 536,543,559–564,566–570,572–582,586–588,590,596] |

| | | | | | | | | | |
|---|---|---|---|---|---|---|---|---|---|
| | | | | | | | | ↓ EGFR/PI3K/Akt pathway<br><br>↓ VEGFR-2 regulated<br><br>↓ PI3K/Akt/mTOR<br><br>↓ IL6<br><br>↓ AR | |
| | | **Coumestans** | **Coumestrol** | Soybean<br><br>Legumes<br><br>Brussels sprouts<br><br>Alfalfa<br><br>Spinach | LNCaP<br><br>PC3 | 0 – 100 μM | ↓ Proliferation<br><br>↓ Migration<br><br>↓ Tumor progression<br><br>Modulation of glucose metabolism | ↑ p53<br><br>↑ Caspase-3 and caspase-9<br><br>↑ DNA damage<br><br>↓ GSK-3<br><br>↓ Akt<br><br>↑ ERK ½<br><br>↓ HIF$\alpha$ | [416,610,611] |
| | **Non-Flavonoids** | **Stilbenoids** | **Resveratrol** | Red grapes<br><br>Peanuts | LNCaP<br><br>DU145<br><br>PC3 | 1 - 150 μM<br><br>Up to 4g/kg or 200 μg/mL | ↓ Cell viability<br><br>↓ Proliferation | ↓ Cyclin D1, B1 and E<br><br>↓ CDK1, CDK4 | [256,627–634,636–647,651] |

| | | | | | | | | | |
|---|---|---|---|---|---|---|---|---|---|
| | | | | | 22RV1<br>MAT-LyLu rat<br>Primary culture from human prostate tumor<br>Primary culture from TRAMP mice<br>LNCaP<br>xenograft in castrated male BALB/cSlc-nu/nu mice<br>Mouse allograft<br>TRAP rat<br>SV-40 Tag rat<br>PTEN KO mice<br>TRAMP mice | | Cell cycle arrest at G1/S phase<br>↓ Survival<br>↑ Apoptosis<br>↓ Migration<br>↓ Invasion<br>Modulation of glucose, glutamine and lipid metabolism<br>↓ PSA levels | ↑ p15, p21 and p27<br>↑ p53<br>↑ FOXO transcription factors<br>↑ BAX, BID, BAK, PUMA, NOXA, BIM, BAD<br>↓ MCL-1, BCL-2, BCL-XL, XIAP, survivin<br>↑ TRAIL receptors<br>↑ FAS system<br>↑ Caspase-7, 9 and 3<br>↑ DNA damage<br>↑ ROS<br>↑ Autophagy<br>↑ E-cadherin | [42,358,624–627,648–650,661] |

| | | | | | | | | | |
|---|---|---|---|---|---|---|---|---|---|
| | | | | | | | | ↓ Vimentin<br>↓ HIFα<br>↑ IGF-1<br>↓ GSK-3<br>↓ PI3K/Akt<br>↓ NFkβ<br>↓ COX-2<br>↓ mTORC1<br>↑ AMPK<br>↑ Hedgehog signaling pathway<br>↓ AR<br>↓ ERα<br>↑ ERβ | |
| **Terpenoids** | **Steroids** | **Sapogenin** | **Diosgenin** | *Dioscorea*<br>*Trigonella*<br>*Costus* | LNCaP<br>DU145<br>PC-3 | 1.4 to 100 μM | ↓ Cell viability<br>↓ Proliferation | ↓ Cyclin D1<br>↓ Bcl-2<br>↓ Bcl-xL | [701,702,704,705,709,711,712] |

| | | | | | | | | | |
|---|---|---|---|---|---|---|---|---|---|
| | | | | *Smilax genera* | C4-2<br><br>TRAMP mice<br><br>DU145 xenograft in nude mice | | Cell cycle arrest at G2/M phase<br><br>↑ Apoptosis<br><br>↑ Autophagy<br><br>↓ EMT<br><br>↓ Migration<br><br>↓ Invasion<br><br>↑ Senescence<br><br>↓ Tumor growth | ↓ Mcl-1<br><br>↓ Survivin<br><br>↓ NEDD4<br><br>↑ Caspase- 9<br><br>↑ p53<br><br>↑ p16<br><br>↑ p21<br><br>↓ MMP-2,-7 and 9<br><br>↓ Mdm2<br><br>↓ Vimentin<br><br>↑ E-Cadherin<br><br>↓ COX-2<br><br>↓ VEGF<br><br>↓ PI3K/Akt/mTOR signaling | |

| | | | | | | | | | |
|---|---|---|---|---|---|---|---|---|---|
| | | | | | | | | ↓ ERK phosphorylation<br><br>↓ JNK phosphorylation<br><br>↓ STAT3 phosphorylation<br><br>↓ Src phosphorylation<br><br>↓ JAK2 phosphorylation<br><br>↓ NFkβ<br><br>↓ EGFR<br><br>↓ TGF-β<br><br>↑ UHRF1 degradation | |
